# Quantitative analysis of targeted lipidomics in the hippocampus of APP/PS1 mice employing the UHPLC-MS/MS method

**DOI:** 10.3389/fnagi.2025.1561831

**Published:** 2025-07-07

**Authors:** Shiyu Xiao, Xuemeng Wei, Bing Han, Xiaoqian Shi, Congzhen Wei, Rumeng Liang, Jingna Sun, Zheng Zhang, Zhengang Han, Li Shen

**Affiliations:** ^1^Department of Clinical Laboratory, The First Hospital of Hebei Medical University, Shijiazhuang, Hebei, China; ^2^Department of Computer Science, Johns Hopkins University, Baltimore, MD, United States; ^3^Department of Neurology, The First Hospital of Hebei Medical University, Shijiazhuang, Hebei, China

**Keywords:** Alzheimer’s disease, APP/PS1 mice, hippocampus, targeted lipidomics, UHPLC-MS

## Abstract

**Background:**

Alzheimer’s disease (AD) is marked by the pathological features of amyloid-*β* plaque accumulation, as well as intracellular neurofibrillary tangles formation in the patients’ brain. Aberrant lipid metabolism is increasingly recognized as one of the important contributors to AD.

**Purpose:**

The main goal of this research was to conduct quantitative detection of targeted lipidomics in hippocampal tissue of APPSwe/PS1dE9 mice in order to identify lipid metabolic biomarkers of early-onset AD mice.

**Methods:**

Our approach departs from conventional lipid detection methods, employing a highly accurate quantificational Ultra High Performance Liquid Chromatography Tandem Mass Spectrometry (UHPLC-MS/MS) technique to analyze targeted lipid biomarkers. The innovative method was utilized to detect targeted lipids in the hippocampus of AD and wild-type mice. Statistical method was performed by Student’s *t*-test and multivariate analysis. Differential metabolites were identified through fulfilling the standard of Variable Importance in Projection surpassing one and the significance probability lower than 0.05 thresholds.

**Results:**

Both groups utilized identical methodologies and adhered strictly to standardized treatment protocols. Sphingolipids (SPs), Glycerophospholipids (GPs), Glycolipids, Glycerides (GLs), Sterol Lipids (STs), and Free Fatty Acid (FA) were identified as prominent lipids exhibiting alterations in the hippocampus of AD models. Regarding glycolipid and glycerolipid composition, monogalactosyldiacylglycerols (MGDGs) and Triacylglycerols (TGs) constituted a significant proportion (*p* < 0.05, VIP > 1). Among glycerophospholipids, phosphatidylethanolamines (PEs) and phosphatidylcholines (PCs) emerged as significant constituents (*p* < 0.05, VIP > 1). Furthermore, hexosylceramides (HexCers) and ceramides (Cers) in the AD model’s hippocampus, the prominent sphingolipids in the hippocampus of AD mice, existed as the two primary changed lipid metabolites. The levels of some TGs in GLs and CEs in STs showed a significant elevation (*p* < 0.05, VIP > 1). In contrast, most kinds of MGDGs, HexCers, Cers, PEs and FA (18:2) demonstrated a notable decrease in the hippocampus of AD group (*p* < 0.05, VIP > 1).

**Conclusion:**

The present research represents the important quantitative identification of distinct lipid biomarker profiles within the hippocampal portion of 7.5-month-aged AD mice. It encompasses glycolipid, GLs, GPs, SPs, STs, and FAs using a targeted HPLC-MS method for quantification. These findings suggest potential diagnostic lipid biomarkers in hippocampus of early-onset AD mice related to cellular membrane integrity, atherosclerosis, oxidative stress damage, and inflammation.

## Introduction

Alzheimer’s disease (AD) is a neurodegenerative disorder that make up 60 to 80 percent of total cases of dementia. It is estimated to have an impact on approximately 152 million people worldwide till 2050 (Calsolaro et al., 2019). As the leading neurodegenerative disorder in elderly individuals with progressive cognitive decline, AD is projected to surge in prevalence as the global population ages (de Veij Mestdagh et al., 2022). AD is marked by the pathological features of amyloid-*β* plaque accumulation, as well as intracellular neurofibrillary tangles formation in the patients’ brain (Fan et al., 2021).

More than two decades ago, the coexistence of APPSwe/PS1dE9 gene alterations was discovered in early-onset familial AD, integrating the Swedish-mutated APP gene alongside the PS1 gene lacking exon 9 (Selkoe and Schenk, 2003). The genetically engineered APPSwe/PS1dE9 mice, that concurrently express mutations in the human APP and PS1 genes, serve as an AD animal model, being characterized by a succession of distinct symptoms of AD patients. The characteristic features also encompassed cognitive decline due to disrupted synapses and the formation of Aβ plaques, which mimic the pathophysiology of human AD (Radde et al., 2006; Gengler et al., 2010). The existence of Aβ plaques in the hippocampal tissue of AD mice, which is vulnerable regions involved in cognition serves as a characteristic indicator of pathology related to cognitive deficits (Li et al., 2022). Furthermore, the hippocampus is a critical region for memory function; however, limited research has been devoted to characterizing targeted lipids within the hippocampus of AD mice. In this study, the implementation of a precise targeted lipidomics ensured the reproducibility of all findings. Male subjects were selected to control for the potential influence of estrogen on experimental outcomes. Both experimental groups received intraperitoneal saline administration to replicate the ordinary treatment approach used in mice. Age-related cognitive decline similar to Alzheimer’s patients was observed in APPSwe/PS1dE9 mice, with deficits manifesting at 7 months of age (Xiong et al., 2011). The experimental deadline was set at seven and a half months for mice, stipulating that observable memory impairment and pathological damage on the hippocampus should be evident in the AD group.

A significant proportion of AD patients develop cerebrovascular complications, with disturbances in lipid homeostasis being a primary contributing factor (Arvanitakis et al., 2016; Attems and Jellinger, 2014). This is particularly evident in the imbalances of phospholipids observed in both serum and cerebrospinal fluid (CSF) of AD patients (Farooqui et al., 1988; Koal et al., 2015), as well as lipid disturbances noted in cortical and serum samples of APP/PS1 mice (Yao et al., 2009; Shen L. et al., 2017). High-density lipoprotein (HDL) exerts a protective effect against cerebrovascular amyloidosis in AD transgenic mice, thereby enhancing overall cerebrovascular health (Button et al., 2019). These findings underscore the regulatory role of lipid modifications in maintaining cerebrovascular health. The therapeutic potential of lipids may be attributed to their ability to regulate lipid homeostasis. While lipidomics provides an advanced approach for identifying novel biomarkers for Alzheimer’s disease diagnosis, it remains in its early stages of development and faces challenges such as insufficient databases and technical complexities (Zhang et al., 2020).

Recent research has included many methods for analyzing lipids, including nontargeted lipidomics utilizing UHPLC-MS with exceptional sensitivity and considerable throughput characteristics. The use of this UHPLC-MS/MS technique is widely used to establish diagnoses through the analysis of dried blood spot samples from newborns for both enzyme activity and glycosaminoglycan (GAG) concentration levels in Genetics such as Mucopolysaccharidosis. Its application in the field of neuroscience, especially neurodegenerative diseases such as Alzheimer’s disease, is promising (Stapleton et al., 2020). Mass spectrometry has proven to be a superior method for uncovering novel lipid biomarkers indicative of an Alzheimer’s disease diagnosis in previous experiments (Yao et al., 2009; Zhang et al., 2020).

At the same time, the untargeted lipidomics were non-absolute quantification. The reproducibility of lipid profiles in the same samples of AD mice across different experiments poses a significant challenge in nontargeted lipidomics analysis. The quantification method for targeted metabolites ensures data accuracy by repeating all experimental details in the quantification process. The lipid results in our research were obtained using their respective standard curves to determine the correlation between variables including the signal intensity and the concentration of analytes. The lipid concentration can be quantified at μg per g of tissue within a linear detection range in targeted lipid analysis. The concentrations of untargeted metabolites, represented by different compounds, cannot be directly inferred from the relative intensities of peaks by LC-MS or GC-MS. Therefore, it is advisable to employ targeted UHPLC-MS for lipid concentration analysis to ensure the precision and reproducibility of findings (Alseekh et al., 2021).

The main goal of this research was to detect lipid biomarkers’ levels of the hippocampal tissue in 7.5-month-aged APP/PS1 mice, with the aim of diagnosing AD of APP/PS1 mice in the early stage of disease and uncovering underlying mechanisms in terms of lipid balance disorders. The lipidomics metabolomics presented herein provides a comprehensive atlas of the lipidomic landscape, making it a valuable resource for exploring the mechanisms underlying hippocampal lipid imbalance in mice with early-onset Alzheimer’s disease.

We also aim to utilize the findings of this research to investigate any therapeutic mechanism of lipids in the hippocampal tissue of APP/PS1 mice within the context of pharmacological experiments where drugs dissolved in saline are administered via intraperitoneal injection as a preventive treatment in APP/PS1 mice.

## Methods

### Animals and administration

The Animal Ethics Committee at the First Hospital of Hebei Medical University (License Number: 20221230) authorized all the protocols involving animals. APPswe/PS1dE9 mice, bred on a C57BL/6 J mouse strain, co-express two genes derived from a chimeric APP gene that possesses the Swedish mutations and the PS1 gene that exhibits a deletion in exon nine (Jankowsky et al., 2004).

Male APPswe/PS1dE9 and C57BL/6 J mice were sourced from SPF Biotechnology Co. Ltd. (Beijing, China). The experimental cohort consisted of 3.5-month-old mice (SCXK Beijing 2019-0010) at the commencement of the trial. All the mice received normal saline (135 mg/kg) through intraperitoneal injection for 3.5 month.

The study utilized standard Makrolon cages with sawdust bedding for housing the mice. The cages enriched with unrestricted access to nourishment and hydration under controlled environmental conditions, with a temperature maintained at 22 ± 2°C and a humidity range at 55 ± 10 percent. The dark–light succession extended from 7:00 pm to 7:00 am, with lights on at 7:00 am, encompassing twelve hours.

### Experimental groups and the procedure for hippocampus tissue preparation and extraction of lipid metabolites

Many drugs used to treat AD patients are formulated in concentrated dosage forms (Huang et al., 2018; Zhou et al., 2019). When these drugs are utilized in animal pharmacological experiments, they must be dissolved in saline or other solvent for administration. In pharmacological studies involving AD animal models, intraperitoneal injection is commonly employed to ensure accurate drug dosing. However, to date, few studies have systematically investigated the mechanisms underlying hippocampal lipid alterations in experiments where drugs are dissolved in saline and administered via intraperitoneal injection to AD mice. Through this experiment, we aim to provide baseline hippocampal lipid profiles for both negative and positive control mice, which will serve as a reference for similar future studies. Therefore, this study was designed with two groups: the APP/PS1 group including APPswe/PS1dE9 mice and the WT control group containing C57BL/6 J mice. Each group consisted of six male mice, and all mice received identical doses of intraperitoneal saline injections.

The daily treatment had been administered for 3.5 months, commencing at 3.5 months in mice and adapting to the environment for 1 week. The mice were euthanized 7 days after the conclusion of the treatment at the age of 7.5-month-old. All the mice were fasted for 12 h prior to anesthetization with isoflurane anesthesia. Brains were excised, and then the hippocampal tissues in two experimental groups were promptly dissected on the ice. The tissues were then stored at −80°C for analysis of lipid metabolites.

The hippocampal tissues were weighed using the ATY124 analytical balance (Shimadzu Corporation, Kyoto, Japan). For each hippocampal sample, 10 ± 0.8 mg of tissue was precisely weighed and diluted with 400 μL of water in an EP tube (concentration: 10 mg/400 μL). The vortex procedure was conducted for 60 s, followed by homogenization of the samples using magnetic beads at a frequency of 45 Hz for 4 min. The homogenate samples were immersed in mixture of ice and water and sonicated for 5 min with PS-60AL 15 L ultrasonic washing machine (Shenzhen Leaderbang Electronics Co., Ltd., Shenzhen, Guandong, China). The homogenate underwent a 5 min sonication process utilizing the same ultrasonicator. The repetition of the homogenization and sonication procedure was carried out thrice in a single cycle. Subsequently, 20 μL hippocampal homogenate was diluted with 180 μL of water.

Lipids were extracted from the hippocampal tissue homogenate with an extraction solution (480 μL, MTBE: MeOH = 5:1) comprising a lipid internal standard. The solution containing the lipid internal standard was gently agitated for 1 min, followed by sonication in a mixture of ice and water for a period of 10 min. After centrifugation (3,000 rpm, 15 min) at a temperature of 4°C, a supernatant volume of 250 μL was obtained. Subsequently, the remaining sediment was mixed with an equal volume of MTBE (250 μL). Afterward, the experimental procedure encompassed vortexing, sonicating, and centrifuging. Subsequently, an additional 250 μL of supernatant was extracted. The supernatants were subsequently pooled and subjected to vacuum concentration at 37°C. The dried samples were rehydrated with 200 μL of a resuspension liquid (comprising of DCM, MeOH, and H_2_O in a ratio of 60:30:4.5). Subsequently, the rehydrated samples underwent rotational agitation for 30 s and then sonicated (10 min) within mixture of ice and water. Following this, the solution was subjected to centrifugation (12,000 rpm, 4°C) for 15 min using microcentrifuge.

After the completion of the experimental procedure, a precise volume of 35 μL liquid portion was carefully transferred and prepared for subsequent UHPLC-MS/MS analysis. Simultaneously, quality control was meticulously created by combining equal volumes of liquid portion derived from each sample.

A comprehensive description of all the experimental procedures is provided in Supplementary Figure S1.

### UHPLC-MS/MS analysis

The separation process of Ultra-high Performance Liquid Chromatography (UHPLC) was carried out utilizing a SCIEX ExionLC UPLC System (SCIEX, Flamingham, MA, United States). The chromatographic column used was an ACQUITY UPLC HSS T3 column (2.1 mm × 100 mm, 1.8 μm) from Waters Corporation (Milford, MA, United States). The mobile phase A comprised 40% H_2_O, 60% CH_3_CN, 10 mmol/L HCOONH_4_. The mobile phase B includes a mixture comprising 10% acetonitrile, 90% isopropanol, and ammonium formate at ten mmol/L concentration. The chromatographic column’s temperature was reserved at 45°C, and the autosampler temperature remained constant at 6°C. For analysis purposes, only four μL of the sample was injected into the system. Detailed information regarding the specific mobile phase conditions employed in liquid chromatography can be found in Table 1.

**Table 1:** Enhanced mobile phase parameters in liquid chromatography.

Time (min)	Flow velocity (μL/min)	A%	B%
0	300	80	20
1	300	80	20
4	300	40	60
15	300	2	98
16	300	2	98
16.01	300	80	20
18	300	80	20

In our research, the SCIEX QTRAP 6500 Plus mass spectrometer (SCIEX, Flamingham, MA, United States) was employed to analyze. The multiple response monitoring (MRM) modes were utilized for mass spectrometry analysis during data acquisition. The ion source parameters were typically configured as follows: Ion Spray Voltage was set to +5,500/−4,500 V; Curtain Gas pressure was maintained at 40 psi; Temperature was controlled at 350°C; Ion Source Gas One and Ion Source Gas Two were both adjusted to 50 psi, and DP (differential pressure) ranged within ±80 V.

Our LC-MS-based strategies have high accuracies, and the LOQ reach down to 0.002 μg/mL and the LOQ reach down to 0.006 μg/mL. For the quality control samples, the coefficient of variation of the lipid normalized area was less than 30% and the retention time was less 5%, indicating the stability of the instrument.

The UHPLC separation was carried out using a SCIEX ExionLC series UHPLC System. The MRM parameters for each of the targeted analytes were optimized using flow injection analysis, by injecting the standard solutions of the individual analytes, into the API source of the mass spectrometer. Several of the most sensitive transitions were used in the MRM scan mode to optimize the collision energy for each Q1/Q3 pair. Among the optimized MRM transitions per analyte, the Q1/Q3 pairs that showed the highest sensitivity and selectivity were selected as “quantifier” for quantitative monitoring. The additional transitions acted as “qualifier” for the purpose of verifying the identity of the target analytes.

### The procedures for data preprocessing

The Biobud-v2.1.4.1 and SCIEX Analyst Software (SCIEX, Flamingham, MA, United States) was employed to quantificate the targeted lipids. The precise content of each lipid, corresponding to the internal standard (IS), was determined by evaluating the peak intensity and utilizing the predetermined concentration of a corresponding internal reference within the same lipid class (SPLASH LIPIDOMIX Mass Spec Standard, AVANTI).

The precise content of each lipid, corresponding to the internal standard (IS), was determined by evaluating the peak intensity and utilizing the predetermined concentration of a corresponding internal reference within the same lipid class. Fifty-eight internal standards were used to quantify lipids, covering 16 lipid classes. All internal standards were obtained from Sigma-Aldrich Trading Co. Ltd. (Merck KGaA, Darmstadt, Germany). The 16 lipid classes included 9 triacylglycerols (TGs), 3 cholesteryl esters (CEs), 5 ceramides (CERs), 5 diacylglycerols (DGs), 1 free fatty acid (FA), 2 hexosylceramides (HexCers), 2 lysophosphatidylcholines (LPCs), 3 lysophosphatidylethanolamines (LPEs), 2 lysophosphatidylserines (LPSs), 5 phosphatidylcholines (PCs), 4 phosphatidylethanolamines (PEs), 5 phosphatidylglycerols (PGs), 5 phosphatidylinositols (PIs), 2 phosphatidylserines (PSs), and 5 sphingomyelins (SMs).

The CentWave algorithm was used for peak detection with the MS/MS spectrum, and lipid identification was achieved through a spectral match using LipidBlast library. Absolute quantification was calculated by normalizing the peak area of each metabolite with respect to the area of the internal standards (IS) and by using standard curves (Sun et al., 2022; Smith et al., 2006).

### Lipid raw data preprocessing

From the original dataset comprising 3 QC and 12 samples, 441 peaks were left after noising filtering. To enhance data analysis, it was necessary to filter the peaks for noise removal (Dunn et al., 2011). Noise filtering retained only those features with ≤50 percentage missing values within or across groups and all QC samples’ RSD < 30%. Missing values were imputed using half of the smallest detected value (Wei et al., 2018). After preprocessing, a total of 441 peaks remained. The absolute lipid content in the samples was determined using internal standards, and the resulting dataset (compound name, sample name, concentration) was imported into SIMCA 16.0.2 (Sartorius Stedim Data Analytics AB, Umea, Sweden) for multivariate analysis.

Firstly, we identified a potential separation trend between the WT control group and APP/PS1 group through PCA analysis. After PCA analysis, the orthogonal projection of latent structure-discriminant analysis (OPLS-DA) was used to screen the significance metabolites. Then a sevenfold cross-validation was performed to calculate the values of *R*^2^ and Q^2^, where *R*^2^ represents the goodness of fit and Q^2^ represents the goodness of prediction. The identification of differentially expressed metabolites was performed by the variable importance in projection (VIP) values (VIP > 1) of OPLS-DA combined with the Student’s *t*-test (*t*-test) (*p* ≤ 0.05).

### Multivariate analysis of lipid data: orthogonal projections to latent structures-discriminant analysis

The data were then normalized and subjected to logarithmic transformation to reduce noise and variance. OPLS-DA was put to be used to distinguish between groups and identify considerably altered metabolites. By removing orthogonal components unrelated to the classification variable, OPLS-DA provided more robust insights into understanding of lipid disparities among groups, even with small sample sizes (Wiklund et al., 2008).

The UV (Unit Variance Scaling) formatting, mean-centered process, and LOG conversion were performed employing SIMCA software. Initially, the modeling analysis was conducted according to the first principal component. The quality and validity of the OPLS-DA model were assessed using seven-fold cross-validation and assessed based on *R*^2^Y (model interpretability) and Q^2^ (predictive ability), separately. A 200-times permutation test confirmed the validity of the model. These scatter plots from OPLS-DA scores were utilized to detect group differences in coefficient of variation.

### Lipid metabolite screening

Our research employed two statistical analysis methods to filter the observed differences. Simultaneously, considering the outcomes of Student’s *t*-test and multivariate statistical analysis enables us to draw comprehensive conclusions from diverse perspectives, thereby avoiding false positive errors. We combined Student’s *t*-test and multivariate analyses to discover differential lipids. This dual approach minimized false positives and potential model overfitting, ensuring conclusions that are reliable.

Firstly, we employed volcano plot analysis to demonstrate the primary down-regulated and up-regulated lipid metabolites. Subsequently, the percentage of altered lipids was illustrated in a donut chart for both groups. Finally, we utilized a box plot to present the representative differential substances in detail and a heatmap to analyze the correlation of lipids in the hippocampus of AD mice.

### Statistical analysis

Statistical evaluation combines Student’s *t*-test and multivariate analysis assessments (SIMCA 16.0.2). Error bars represent SEM. Lipids were considered significantly changed if VIP exceeded 1 and *p*-value was lower than 0.05. The degree of correlation was evaluated using the Pearson method as a statistical measure.

## Results

### The results of quality control

**Figure 1:**
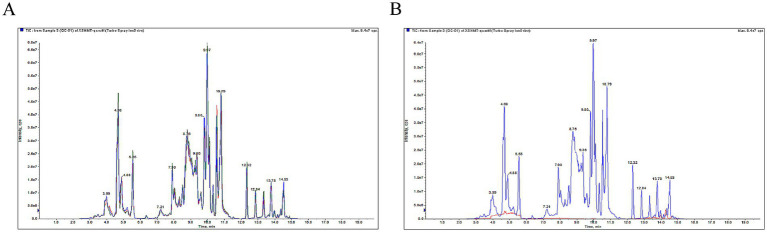
**(A)** TIC diagram of all QC samples. **(B)** The TIC diagram of sample “QC-01” and blank sample.

Quality control (QC) ensured stable instrument performance and reliable detection of standards. The consistent overlap in total ion current (TIC) retention times and peak areas for QC samples (Figure 1A) confirmed satisfactory system stability.

Analyzing blank samples can help investigate substance residues in the detection process. As illustrated in Figure 1B, no significant interference peak was observed in the blank sample compared to sample “QC-01,” indicating minimal cross-contamination and effective residue control.

To validate the peak, peak area of sample should be larger than 3*STD of area of blank, and the RSD of all QC samples should be less than 30%.

### Multivariate analysis results

**Figure 2:**
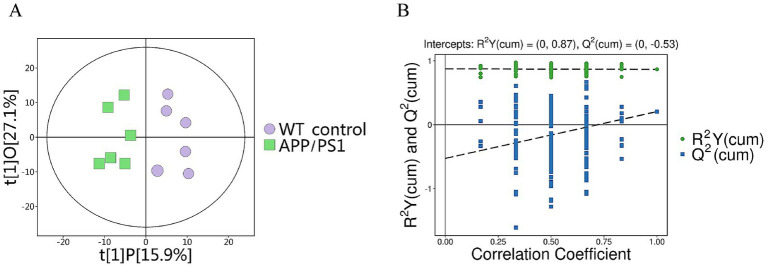
**(A)** OPLS-DA score plot for group APP/PS1 versus WT control. The OPLS-DA score plot demonstrated that the samples were dispersed into two groups (*n* = 12). The data for the WT control group are shown in purple, and the data for the APP/PS1 group are shown in green. **(B)** OPLS-DA permutation test for APP/PS1 versus WT control group. The green dot stands for the *R*^2^Y value acquired from the permutation test, the blue square dot indicates the Q^2^ value obtained from the permutation test, and the two dashed lines, respectively, represent the regression lines of *R*^2^Y and Q^2^.

Using the UHPLC-MS/MS method, we successfully detected 441 peaks. The PCA score plot indicates that all samples lie within the 95% confidence interval, as defined by Hotelling’s T-squared ellipse (Supplementary Figure S2). OPLS-DA was then applied to the lipidomic data to minimize confounding variables and clarify group differences. Figure 2 shows the OPLS-DA score plot distinguishing APP/PS1 from WT controls. The separation along the predictive component (x-axis) highlights significant differences between the groups, with minimal within-group variability along the orthogonal component (y-axis). All data points lay within the 95% confidence range defined by Hotelling’s T^2^ ellipse. Cross-validation yielded *R*^2^Y = 0.867 and Q^2^ = 0.205. These values reflected the model’s effectiveness and predictability in explaining categorical variables. Furthermore, the validity of the observed differences between the two groups shown in Figure 2B was confirmed through 200 times of permutation tests. In these tests, the horizontal axis represents the permutation order of the Y variable, with “1” indicating the original model’s arrangement. The vertical axis displays *R*^2^Y and Q^2^ values.

### Lipid composition analysis

**Figure 3:**
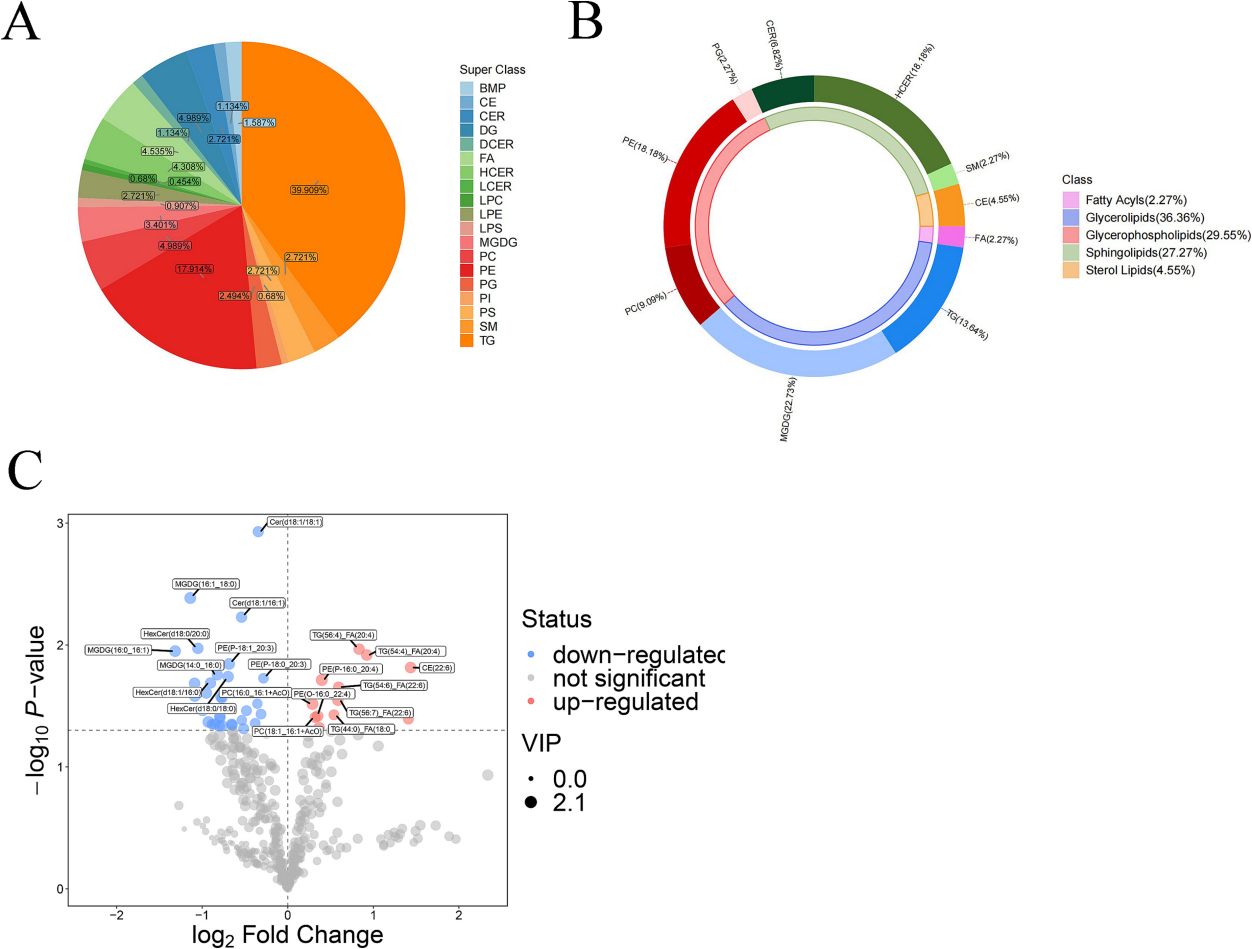
**(A)** Pie chart illustrating the distribution of lipid compositions by the superclass. **(B)** Donut chart for group APP/PS1 vs. WT control. **(C)** Volcano plot of lipid metabolites comparing group APP/PS1 with WT control. Red signifies lipids that are significantly up-regulated; blue indicates lipids that are significantly down-regulated; gray denotes lipids that showed no significant changes.

As illustrated in Figure 3, the lipid composition pie chart analyzed the relative abundance of different lipid types detected in all samples. In the hippocampus, five major classes of lipids were identified, namely glycerolipids (48.30%), glycerophospholipids (34.69%), sphingolipids (11.34%), fatty acyls (4.54%), and sterol lipids (1.13%). Notably, free fatty acids (FA) constituted the predominant constituents within the fatty acyl class, while cholesterol esters (CE) dominated the composition of sterol lipids.

Hexosylceramides (HCER), ceramides (CER), sphingomyelins (SM), dihydroceramides (DCER), and lactosylceramides (LCER) accounted for 4.31, 2.72, 2.72, 1.13, and 0.45%, respectively, of the total lipid content. Phosphatidylethanolamines (PE) and phosphatidylcholines (PC) were the two major constituents of glycerophospholipids, contributing to 17.91 and 4.99%, respectively, of the overall lipid composition. Additionally, as a minor fraction within glycerophospholipids, phosphatidylserine (PS), lysophosphatidylethanolamines (LPE), phosphatidylglycerols (PG), bis(monoacylglycero)phosphate (BMP), lyso-phosphatidylserine (LPS), phosphatidylinositol (PI) and lysophosphatidylcholines (LPC) constituted 2.72, 2.72, 2.49, 1.59, 0.91, 0.68, and 0.68% of the entire lipid composition. Furthermore, triacylglycerols (TGs), diacylglycerols (DGs), and monogalactosyldiacylglycerol (MGDG) comprised a substantial proportion of glycerolipid content at 39.91, 4.99, and 3.40%, respectively.

### Differential lipid metabolites analysis

Forty-three lipid metabolites showed significant differences between the APP/PS1 group and the WT control group, with the top twenty highlighted in Table 2. Key alterations of lipids in the hippocampus of AD models are described below.

**Table 2:** Differences were observed in the representative lipid metabolites revealed in the hippocampus of APP/PS1 group.

Peak	Lipid metabolite	RT (min)	Q1 (m/z)	Q3 (m/z)	VIP	*P*-value	Fold change
1	CE(22:4)	14.54	718.60	369.40	1.29	0.049	1.78
2	CE(22:6)	13.90	714.60	369.40	1.86	0.015	2.70
3	HexCer(d18:0/20:0)	9.30	758.70	266.26	1.80	0.011	0.48
4	HexCer(d18:1/16:0)	7.78	700.70	264.26	1.76	0.020	0.53
5	MGDG(16:0_16:0)	8.80	789.60	255.23	1.85	0.025	0.52
6	MGDG(16:0_16:1)	8.11	787.60	255.23	1.95	0.011	0.40
7	MGDG(16:0_18:1)	8.82	815.60	255.23	1.96	0.026	0.47
8	MGDG(16:1_18:0)	8.87	815.60	253.22	2.05	0.004	0.45
9	MGDG(18:0_18:1)	9.60	843.60	283.26	1.89	0.034	0.50
10	MGDG(18:1_18:1)	8.90	841.60	281.25	1.86	0.021	0.47
11	MGDG(18:1_22:0)	10.97	899.70	281.25	1.96	0.043	0.53
12	PC(16:0_20:3 + AcO)	8.64	842.60	305.25	1.97	0.048	1.29
13	PE(P-16:0_20:4)	8.40	722.50	303.23	2.11	0.019	1.32
14	PG(20:1_24:1)	9.79	885.70	309.28	1.36	0.045	0.54
15	TG(44:0)_FA(18:0)	13.11	768.70	467.41	1.51	0.037	1.45
16	TG(54:4)_FA(20:4)	13.90	900.80	579.50	1.85	0.012	1.90
17	TG(54:6)_FA(22:6)	13.30	896.80	551.50	1.75	0.022	1.51
18	TG(56:4)_FA(20:4)	14.30	928.80	607.50	1.81	0.011	1.78
19	TG(56:7)_FA(22:6)	13.30	922.80	577.50	1.80	0.028	1.50
20	TG(58:8)_FA(22:6)	13.31	948.80	603.50	1.55	0.040	2.66

Q1: Mass-charge ratio of parent ion Q3: Mass-charge ratio of fragment ion.

Figure 3B (donut chart) summarizes the major altered lipid classes in the APP/PS1 hippocampus. Glycerolipids, glycerophospholipids, and sphingolipids were most affected, representing 36.36, 29.55, and 27.27% of all altered metabolites, respectively. Regarding glycerolipid composition, MGDG and TG constituted a significant proportion of 22.73 and 13.64%, respectively. Among glycerophospholipids, phosphatidylethanolamines (PE), phosphatidylcholines (PC), and phosphatidylglycerols (PG) emerged as significant constituents, accounting for 18.18, 9.09, and 2.27% of the overall altered lipid composition. Furthermore, hexosylceramides (HCER) and ceramides (CER), as primary changed constituents of sphingolipids in the hippocampal region of AD mice, accounted for 18.18 and 6.82% respectively, of the total lipid type with observed changes in content.

The volcano plot graph visualizes the most prominent differential lipid metabolites. As illustrated in Figure 3C, each data point stands for a kind of specific lipid substance, with the x-axis representing the log_2_ fold change and the y-axis displaying the –log_10_ (*p*-value). Moreover, point size reflects its VIP value derived from the OPLS-DA model, with more significant data points indicating higher VIP values.

On the right side of the volcano plot, several Triacylglycerols (TG) species- TG(56:4)_FA(20:4), TG(56:7)_FA(22:6), TG(54:4)_FA(20:4), TG(54:6)_FA(22:6), and TG(44:0)_FA(18:0)-exhibited up-regulation in the hippocampus of APP/PS1 mice. CE (22:6) levels were also elevated. In addition, certain phosphatidylethanolamines (PE(O-16:0_22:4), PE(P-16:0_20:4)) and phosphatidylcholines (PC(18:1_16:1 + AcO), PC(16:0_16:1 + AcO)) increased in the AD model hippocampus.

Conversely, the left side of the volcano plot revealed decreased levels of multiple MGDG species (MGDG (16:1_18:0), MGDG (16:0_16:1), MGDG (14:0_16:0)) in the hippocampus of APP/PS1 mice. Several HexCers (HexCer(d18:0/20:0), HexCer(d18:0/18:0), HexCer(d18:1/16:0)) and Cers (Cer(d18:1/18:1), Cer(d18:1/16:1)) were also downregulated. Additionally, certain PEs (PE(P-18:0_20:3), PE(P-18:1_20:3)) showed decreased concentrations.

**Figure 4:**
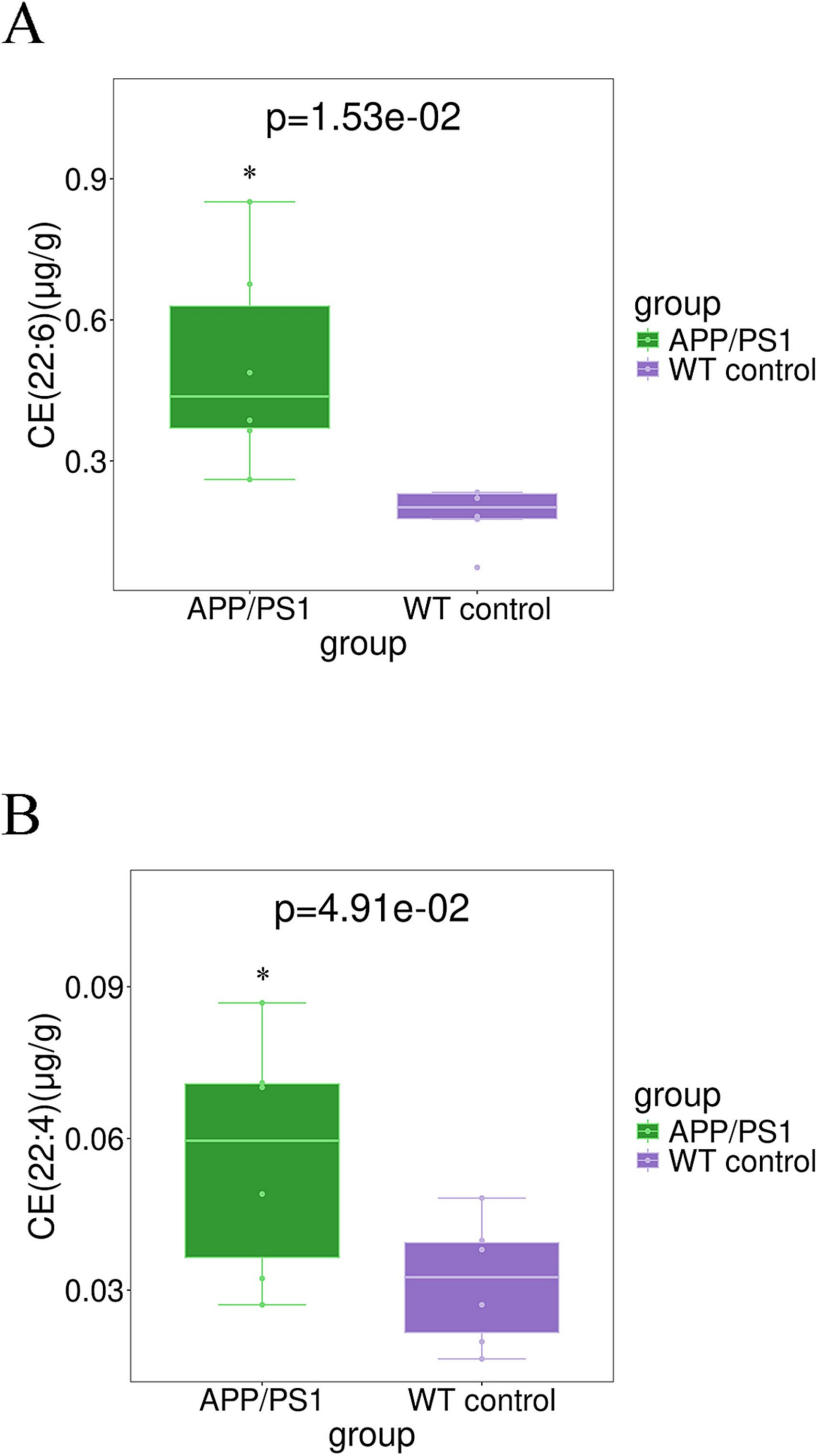
Boxplot analysis of **(A)** CE (22:6) and **(B)** CE (22:4) in the hippocampal tissue for group APP/PS1 vs WT control.

**Figure 5:**
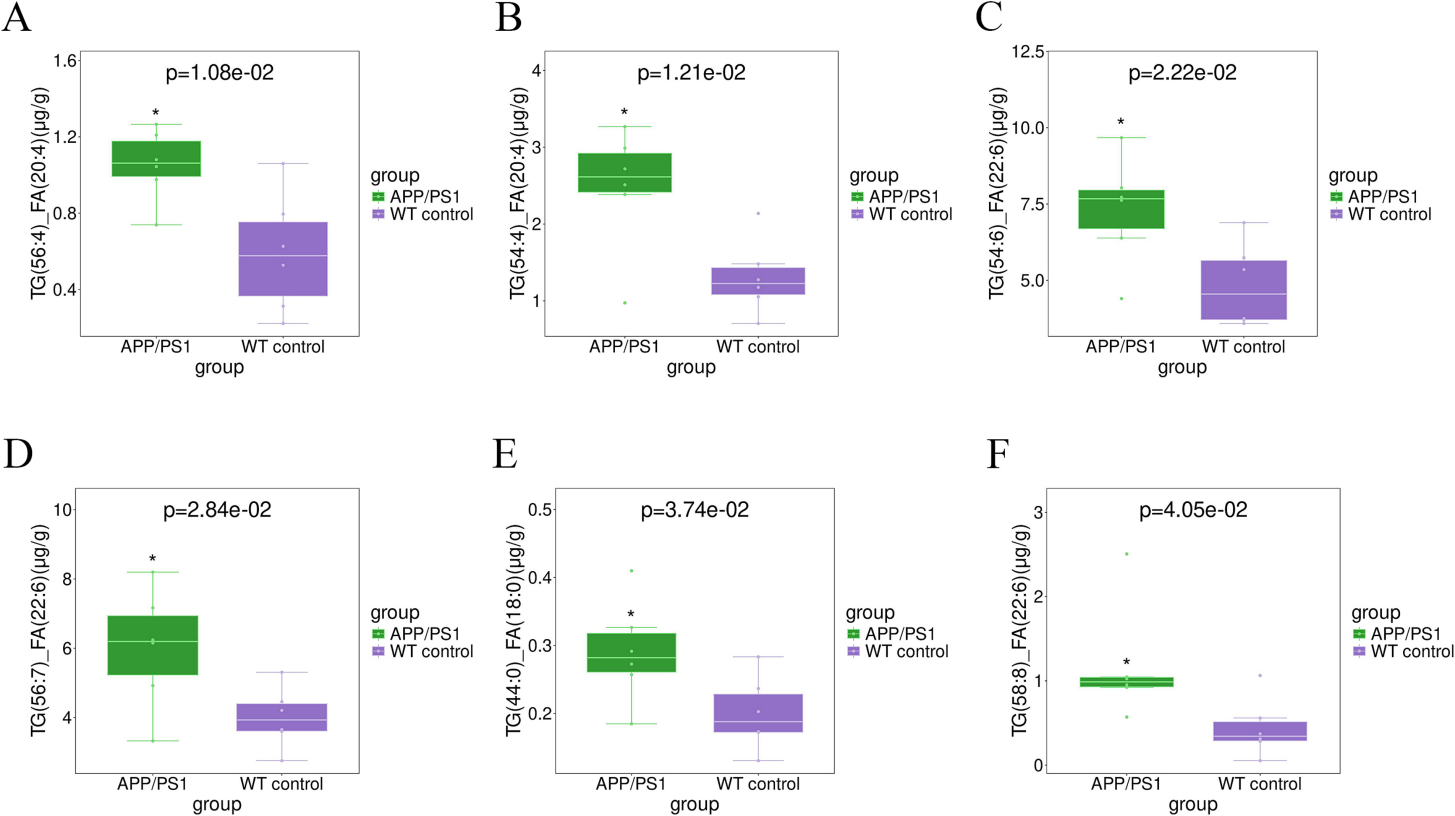
Boxplot analysis comparing the levels of various TAGs in hippocampal tissue between the APP/PS1 and WT control mice. **(A)** TG(56:4)_FA(20:4) (*p* = 0.011); **(B)** TG(54:4)_FA(20:4) (*p* = 0.012); **(C)** TG(54:6)_FA(22:6) (*p* = 0.022); **(D)** TG(56:7)_FA(22:6) (*p* = 0.028); **(E)** TG(44:0)_FA(18:0) (*p* = 0.037); **(F)** TG(58:8)_FA(22:6) (*p* = 0.040).

**Figure 6:**
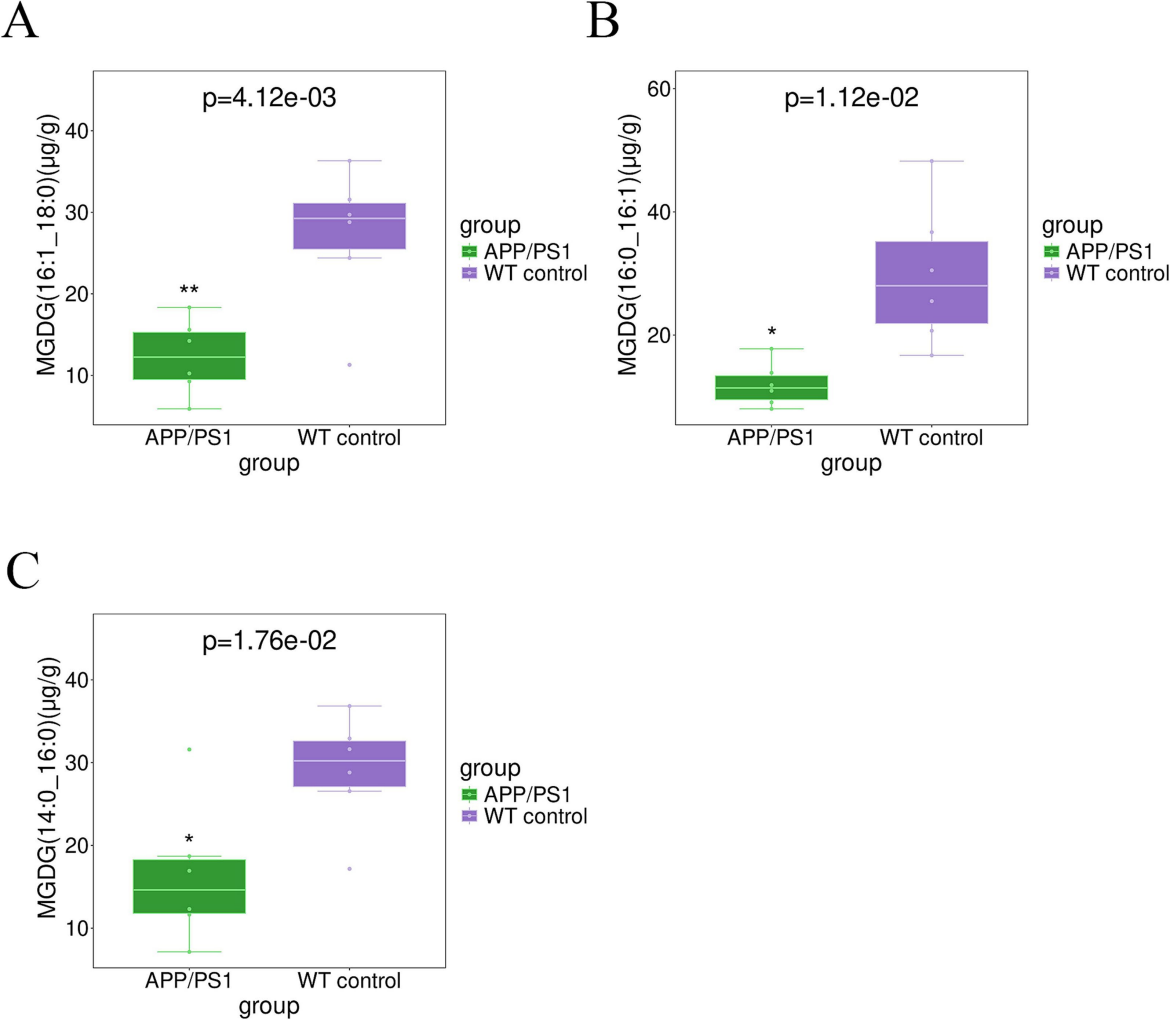
The boxplot analysis demonstrated a remarkable reduction in the levels of MGDGs in the hippocampus of APP/PS1 group in contrast to WT control, suggesting significant changes. **(A)** MGDG (16:1_18:0); **(B)** MGDG (16:0_16:1); **(C)** MGDG (14:0_16:0).

**Figure 7:**
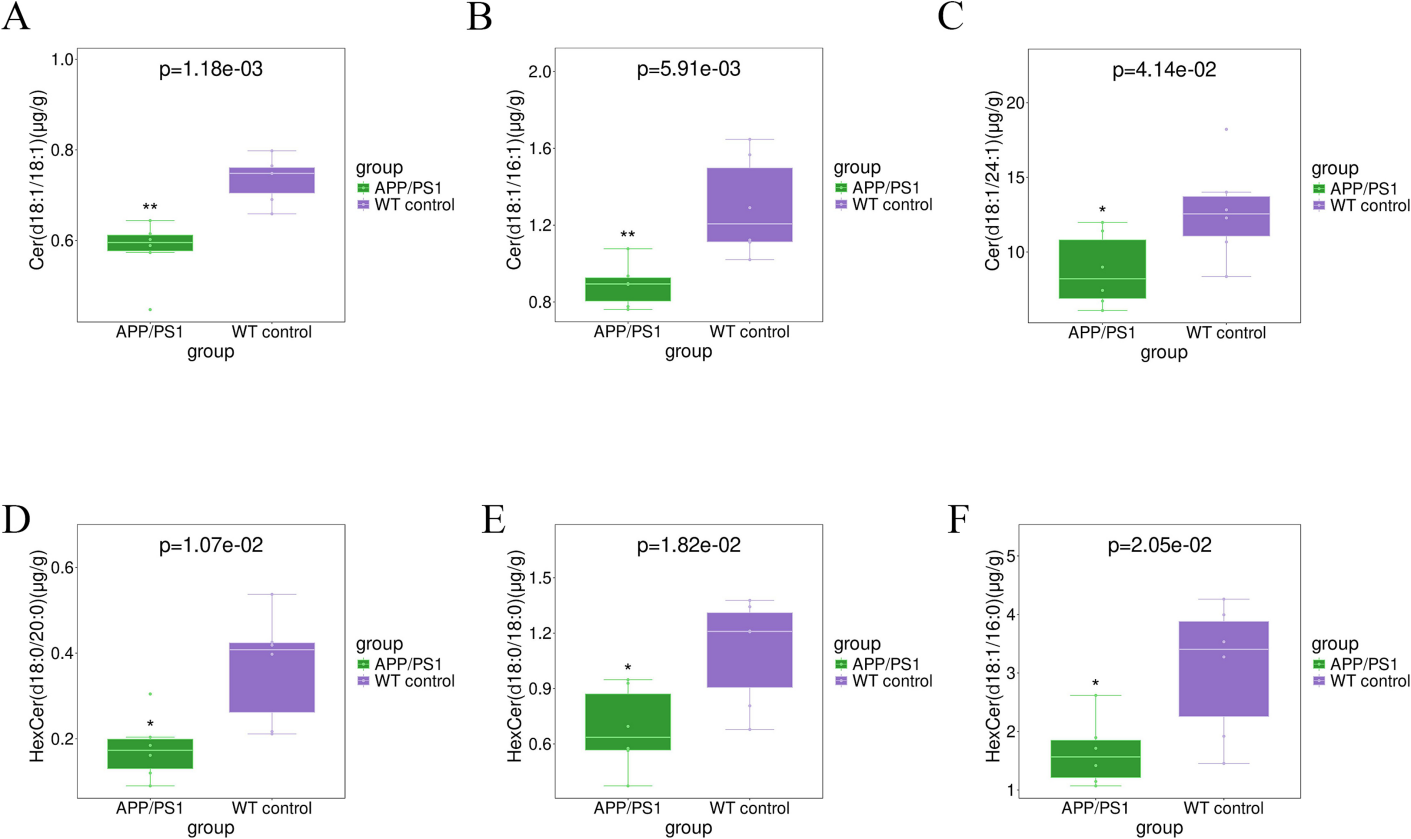
Boxplot analysis of CER and HCER existing the most difference in the hippocampus tissue for group APP/PS1 vs WT control. **(A)** Cer(d18:1/18:1); **(B)** Cer(d18:1/16:1); **(C)** Cer(d18:1/24:1); **(D)** HexCer(d18:0−/20:0); **(E)** HexCer(d18:0/18:0); **(F)** HexCer(d18:1/16:0).

The elevated level of CE (22:6) (*p* = 0.015) and CE (22:4) (*p* = 0.049) in the hippocampal tissue of APPswe/PS1dE9 mice, as depicted in Figure 4, surpassed that observed in WT control mice. The levels of TG(56:4)_FA(20:4) (*p* = 0.011), TG(54:4)_FA(20:4) (*p* = 0.012), TG(54:6)_FA(22:6) (*p* = 0.022), TG(56:7)_FA(22:6) (*p* = 0.028), TG(44:0) _FA(18:0) (*p* = 0.037), and TG (58:8)_FA(22:6) (*p* = 0.040), in the hippocampal tissues of AD mice exhibited significant elevation, as depicted in Figure 5. Conversely, majority of MGDG decreased in hippocampus of AD mice as shown in Table 3. The levels of MGDG(16:1_18:0), MGDG(16:0_16:1), and MGDG(14:0_16:0) demonstrated a notable reduced with *p* values of 0.004, 0.011, and 0.018, respectively, in Figure 6. Table 3 highlights HexCers and Cers as key sphingolipids altered in APP/PS1 mice. As illustrated in Figure 7, Cer(d18:1/18:1), Cer(d18:1/16:1), and Cer(d18:1/24:1) decreased significantly (*p* = 0.001, *p* = 0.006, *p* = 0.041, respectively). Most HexCer species, including HexCer(d18:0/20:0), HexCer(d18:0/18:0), and HexCer(d18:1/16:0), also showed marked reductions. The corresponding *p* values for these changes were 0.011, 0.018, and 0.020, respectively.

**Table 3:** The lipid profiles in hippocampal tissues differ between WT mice and APP/PS1 mice.

No.	Compound name	RT	WT(μg/g)	APP/PS1(μg/g)	*P*-value	Fold change	VIP
1	CE(22:4)	14.541	0.03 ± 0.01	0.06 ± 0.01	0.049	1.78	1.29
2	CE(22:6)	13.904	0.19 ± 0.02	0.50 ± 0.09	0.015	2.70	1.86
2	Cer(d18:1/16:1)	7.907	1.29 ± 0.11	0.89 ± 0.05	0.006	0.69	1.76
3	Cer(d18:1/18:1)	8.7	0.73 ± 0.02	0.58 ± 0.03	0.001	0.79	1.67
4	Cer(d18:1/24:1)	10.804	12.73 ± 1.36	8.77 ± 1.01	0.041	0.69	1.34
5	FA(18:2)	4.2	18.29 ± 0.70	14.73 ± 1.30	0.037	0.81	1.46
6	HexCer(d18:0/18:0)	8.5	1.10 ± 0.12	0.68 ± 0.09	0.018	0.62	1.88
7	HexCer(d18:0/20:0)	9.296	0.37 ± 0.05	0.18 ± 0.03	0.011	0.48	1.80
8	HexCer(d18:0/22:0)	10	1.71 ± 0.26	0.98 ± 0.19	0.046	0.57	1.75
9	HexCer(d18:1/16:0)	7.778	3.07 ± 0.46	1.64 ± 0.23	0.020	0.53	1.76
10	HexCer(d18:1/18:0)	8.5	57.92 ± 7.39	36.91 ± 5.46	0.045	0.64	1.66
11	HexCer(d18:1/20:0)	9.296	16.48 ± 2.39	9.51 ± 1.72	0.039	0.58	1.76
12	HexCer(d18:1/20:1)	9.052	34.49 ± 4.75	20.08 ± 2.87	0.027	0.58	1.79
13	HexCer(d18:1/22:0)	10	82.89 ± 11.92	48.72 ± 9.10	0.046	0.59	1.73
14	MGDG(14:0_16:0)	8.063	28.99 ± 2.77	16.38 ± 3.47	0.018	0.57	1.72
15	MGDG(16:0_16:0)	8.796	257.07 ± 37.20	132.84 ± 28.82	0.025	0.52	1.85
16	MGDG(16:0_16:1)	8.111	29.74 ± 4.69	11.94 ± 1.44	0.011	0.40	1.95
17	MGDG(16:0_18:0)	9.5	101.32 ± 15.77	56.03 ± 11.84	0.045	0.55	1.79
18	MGDG(16:0_18:1)	8.819	145.54 ± 26.25	68.53 ± 13.56	0.026	0.47	1.96
19	MGDG(16:0_20:0)	10.211	47.84 ± 6.48	27.60 ± 5.47	0.038	0.58	1.83
20	MGDG(16:1_18:0)	8.87	27.01 ± 3.52	12.26 ± 1.88	0.004	0.45	2.05
21	MGDG(18:0_18:1)	9.6	61.81 ± 10.86	31.03 ± 6.34	0.034	0.50	1.89
22	MGDG(18:1_18:1)	8.896	29.71 ± 5.10	13.96 ± 2.61	0.021	0.47	1.86
23	MGDG(18:1_22:0)	10.974	3.81 ± 0.60	2.00 ± 0.49	0.043	0.53	1.96
24	PC(16:0_16:1 + AcO)	8.304	145.09 ± 8.05	177.14 ± 9.85	0.030	1.22	2.10
25	PC(16:0_20:3 + AcO)	8.644	30.84 ± 2.66	39.74 ± 2.92	0.048	1.29	1.97
26	PC(18:0_18:0 + AcO)	10.089	69.64 ± 4.98	54.44 ± 3.39	0.030	0.78	1.27
27	PC(18:1_16:1 + AcO)	8.337	17.94 ± 1.22	22.40 ± 1.43	0.039	1.25	1.75
28	PE(O-16:0_22:4)	9.122	19.89 ± 1.32	25.34 ± 1.86	0.038	1.27	1.89
29	PE(P-16:0_16:1)	8.467	3.78 ± 0.36	2.71 ± 0.25	0.035	0.72	1.52
30	PE(P-16:0_20:4)	8.4	35.79 ± 2.57	47.14 ± 3.17	0.019	1.32	2.11
31	PE(P-18:0_16:1)	9.3	10.16 ± 0.78	7.80 ± 0.66	0.044	0.77	1.46
32	PE(P-18:0_20:3)	9.6	31.97 ± 1.20	26.25 ± 1.65	0.019	0.82	1.40
33	PE(P-18:1_18:0)	9.27	18.80 ± 2.41	11.94 ± 1.77	0.045	0.64	1.69
34	PE(P-18:1_18:2)	8.652	8.97 ± 0.82	6.28 ± 0.88	0.049	0.70	1.58
35	PE(P-18:1_20:3)	8.819	50.43 ± 5.84	31.36 ± 2.72	0.014	0.62	1.95
36	PG(20:1_24:1)	9.789	5.48 ± 0.90	2.96 ± 0.62	0.045	0.54	1.36
37	SM(24:0)	10.633	6.57 ± 0.92	3.86 ± 0.51	0.027	0.59	1.65
38	TG(44:0)_FA(18:0)	13.11	0.20 ± 0.02	0.29 ± 0.03	0.037	1.45	1.51
39	TG(54:4)_FA(20:4)	13.9	1.30 ± 0.20	2.47 ± 0.33	0.012	1.90	1.85
40	TG(54:6)_FA(22:6)	13.3	4.84 ± 0.56	7.31 ± 0.72	0.022	1.51	1.75
41	TG(56:4)_FA(20:4)	14.3	0.59 ± 0.13	1.05 ± 0.08	0.011	1.78	1.81
42	TG(56:7)_FA(22:6)	13.304	4.00 ± 0.36	6.00 ± 0.70	0.028	1.50	1.80
43	TG(58:8)_FA(22:6)	13.311	0.44 ± 0.14	1.17 ± 0.28	0.040	2.66	1.55

**Figure 8:**
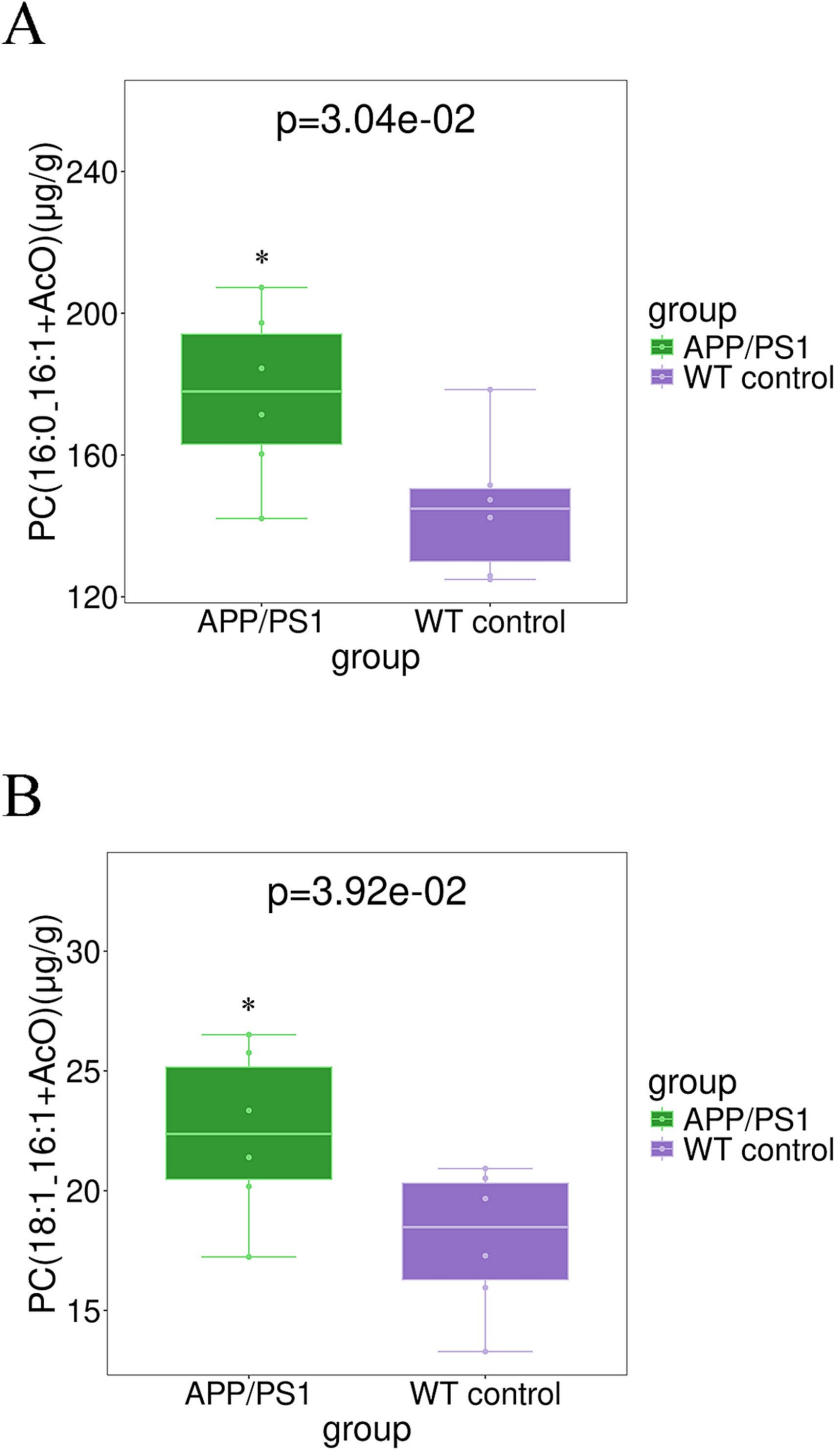
Boxplot analysis of PCs in the hippocampus tissue for group APP/PS1 vs WT control. **(A)** PC (16:0_16:1 + AcO); **(B)** PC (18:1_16:1 + AcO).

As depicted in Figure 8, the levels of hippocampal glycerophospholipid’ main components, such as PC (16:0_16:1 + AcO) and PC (18:1_16:1 + AcO), exhibited a significant increase in APP/PS1 mice, with *p* values of 0.030 and 0.039, respectively.

**Figure 9:**
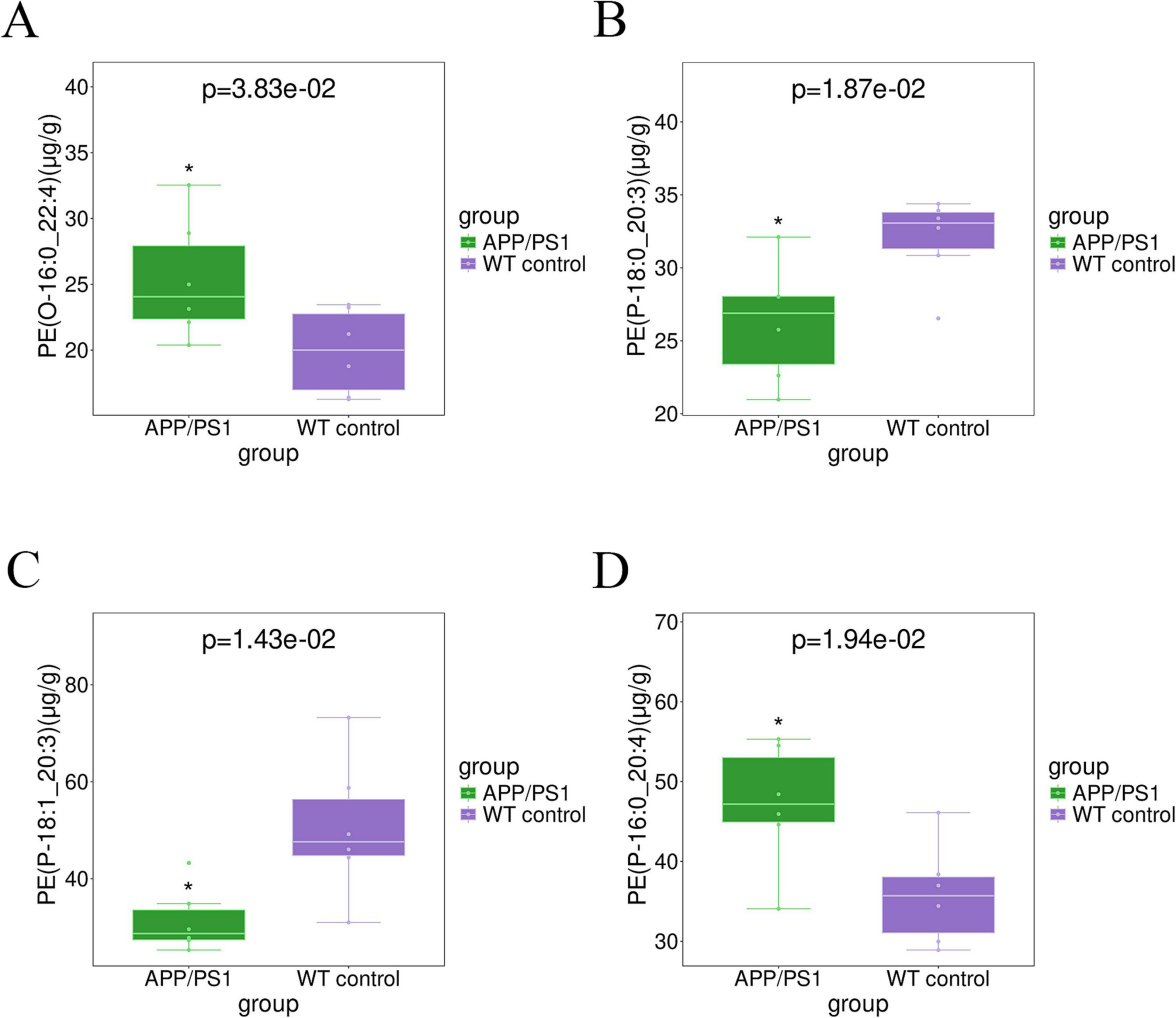
Boxplot analysis of PEs in the hippocampus tissue for group APP/PS1 vs WT control. **(A)** PE(O-16:0_22:4); **(B)** PE(P-18:0_20:3); **(C)** PE(P-18:1_20:3); **(D)** PE(P-16:0_20:4).

As depicted in Figure 9, the levels of PE(O-16:0_22:4) and PE(P-16:0_20:4) in the certain hippocampal phosphatidylethanolamines’ components exhibited a significant increase in APP/PS1 mice, with *p* values of 0.038 and 0.019, respectively. Other species of phosphatidylethanolamines’ components such as PE(P-18:1_20:3), PE(P-18:0_20:3) in Figure 9 and others in Table 3 decreased in the hippocampus of AD mice, with *p* values of 0.014 and 0.019, respectively.

A detailed description of the differential lipid metabolites is presented in Supplementary Figure S1.

### Correlation analysis of differential lipid metabolites

**Figure 10:**
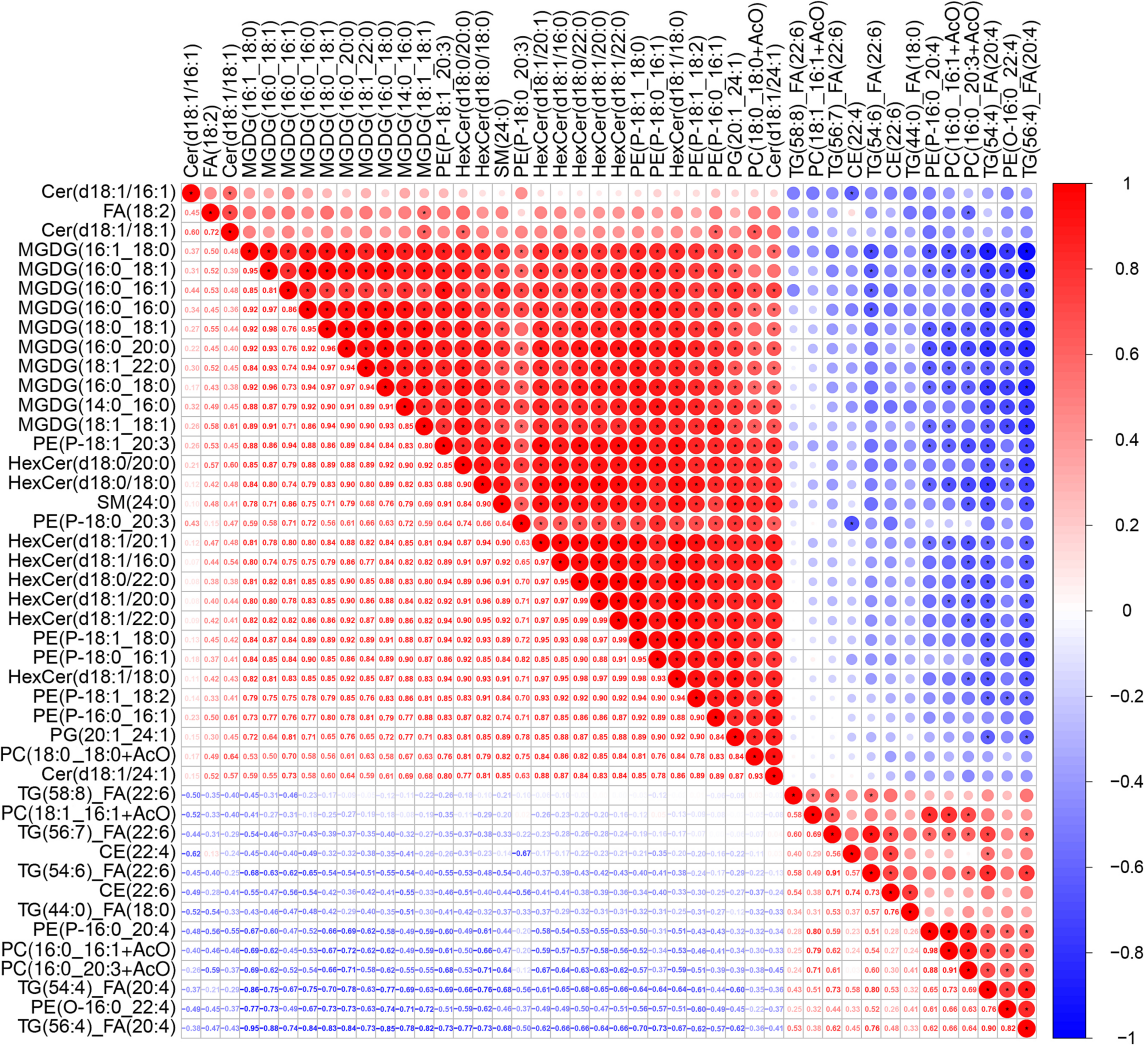
Heatmap depicting the correlation analysis of potential lipid biomarkers in the hippocampus of 7.5-month-old APP/PS1 mice. The two-color heat map visually represents the association between lipids. x-axis and y-axis depict the variations in metabolites among the groups, whereas the color blocks at various locations signify the correlation coefficients between the respective metabolites. Red color denotes a positive association, while blue color signifies a negative association, and darker colors indicate stronger correlations. Significant correlations are marked with an asterisk (*).

A Pearson correlation heatmap (Figure 10) revealed interrelationships and potential synergistic effects among altered lipids in APP/PS1 group contrasted with WT control (Pripp, 2018). The heatmap displays stronger correlations between variables, indicated by correlation coefficients represented with “r” closer to 1 in absolute value. A correlation coefficient (r) greater than 0.8 indicates a high correlation between two types of lipids, while an r value less than 0.3 suggests a low correlation. For values between 0.8 and 0.3, it implies a moderate correlation exists.

Because there was a relationship between lots of lipid metabolites in the APP/PS1 group, we only explored the highly correlated metabolites among the lipids in the hippocampal tissue that showed apparent differences based on the data in the previous section. For example, TG(54:4)_FA(20:4) positively correlated with TG(54:6)_FA(22:6) (r = 0.801) and TG(56:4)_FA(20:4) (r = 0.898), but negatively correlated with MGDG(16:1_18:0) (r = −0.861).

HexCer species (HexCer(d18:0/18:0), HexCer(d18:1/16:0), HexCer(d18:0−/20:0) were strongly correlated with each other. HexCer(d18:0/18:0) correlated positively with Cer(d18:1/24:1) (r = 0.810), MGDG(16:1_18:0) (r = 0.840), and PE(P-18:1_20:3) (r = 0.877). HexCer(d18:0−/20:0) correlated strongly with MGDG(14:0_16:0) (r = 0.817), MGDG (16:1_18:0) (r = 0.854), and PE(P-18:1_20:3) (r = 0.851). HexCer(d18:1/16:0)) also showed high positive correlations with MGDG (14:0_16:0) (r = 0.895), MGDG(16:1_18:0) (r = 0.802), and PE(P-18:1_20:3) (r = 0.886).

Various kinds of MGDGs were generally positively intercorrelated. MGDG (14:0_16:0) correlated with PE(P-18:1_20:3) (r = 0.832), and MGDG (16:0_16:1) also correlated with PE(P-18:1_20:3) (r = 0.938). In contrast, MGDG (16:1_18:0) negatively correlated with TG(56:4)_FA(20:4) (r = −0.947). PC (16:0_16:1 + AcO) and PC (18:1_16:1 + AcO) positively correlated with PE(P-16:0_20:4) (r = 0.983 and r = 0.803, respectively). PE(P-18:1_20:3) also showed positive correlations with various other PE species and multiple HexCer and MGDG metabolites.

## Discussion

Alzheimer’s disease (AD) is defined by progressive cognitive deterioration, linguistic deficiencies, visuospatial abnormalities, and emotional disturbances. Its impact extends beyond patients, placing significant emotional, social, and economic burdens on families and society.

Although extracellular Aβ plaques and neurofibrillary tangles define the core pathology of AD, the disease also involves other lesions including oxidative stress, inflammation, and disrupted membrane lipid metabolism (Selkoe, 2003; Kosicek and Hecimovic, 2013; Maccioni et al., 2001). These lesions predominantly affected the hippocamal tissue and cortex tissue (Maccioni et al., 2001; Braak and Braak, 1991). The destroyed tissue mentioned above are particularly vulnerable in early AD (González-Domínguez et al., 2014d). Subsequent progression of the pathological changes and damage leads to the manifestation of severe clinical symptoms as described above. However, direct investigation on the pathological lesions and subsequent metabolic perturbations in human AD brains of AD patients remains difficult. Because of its simple operation, transgenic mice have also been always used to investigate the performance, pathophysiology (Yokoyama et al., 2022), metabolic perturbations of AD, mimicking certain pathological characteristics such as deposition of Aβ plaques and behavioral deficits observed in AD in recent years (González-Domínguez et al., 2014d; Malm et al., 2011). APP/PS1 mice show early pathology by 4 months and measurable cognitive decline 6 months later in previous research (Malm et al., 2011). In our previous research, the cognitive destruction and lipid disorder occurred in APP/PS1 mice at 7 months old. So, our experimental deadline was set at seven and a half months for APP/PS1 mice, guaranteeing that observable memory impairment and pathological damage on the hippocampus should be evident in the AD group.

Fasting is primarily used as a way of standardizing tests in which case mice are usually fasted for 4 h up to overnight (Jensen et al., 2013). There was no difference in plasma FA in concentration in 16 h fasted mice compared with 4 h fasted mice (Jensen et al., 2013; Heijboer et al., 2005). Previous researcher did not observe any changes in the VLDL, LDL and HDL of the various fractions (TG, FC, PL, TC) in the blood profiles of C57bl6 mice after 24 h starvation (van Ginneken et al., 2007). There were two groups in our experiment. All the mice in both groups were fasted prior to brain harvest. Even if the effect of fasting was present, its impact on lipids would cancel out when comparing lipid levels of hippocampus between the two groups.

The lipid-related anomalies were initially identified in the neuropathology research (Alzheimer et al., 1995). Over time, lipid dyshomeostasis has become a major focus in AD research, with evidence from animal models and clinical evidence linking disrupted lipid metabolism to disease initiation and progression (Zhang et al., 2020; Touboul and Gaudin, 2014; Peña-Bautista et al., 2022). Beyond senile plaques and neurofibrillary tangles, AD brains also accumulate abnormal lipid deposits (“adipose inclusions” or “lipoid granules”) (Foley, 2010). Aberrant lipid metabolism is increasingly recognized as a key contributor to neurodegeneration, including AD (Zhang et al., 2022; Toledo et al., 2017; He et al., 2010; Kao et al., 2020). Extensive lipidomic perturbations are found in the hippocampus and cortex in AD (González-Domínguez et al., 2014d). Interestingly, prior research have demonstrated that a high-fat/glucose diet can induce biochemical alterations associated with increased AD biomarker burden and cognitive impairment, thereby suggesting a detrimental effect of lipids on Alzheimer’s disease (AD) (Kothari et al., 2017; Hill et al., 2019). Previously, we reported elevated LDL-C and reduced HDL-C in the serum from 7-month-old AD mice, along with hippocampal glucose disturbances (Shen L. et al., 2017). At 6 months old, hippocampal lipid of AD mice alterations were mild when assessed by GC-TOF/MS (Han et al., 2017). Subsequent lipidomic analysis unveiled that the differentially expressed plasma lipid metabolites between 9-month-old APP/PS1 and WT mice predominantly encompassed phosphatidylcholines, lysophosphatidylcholines, triglycerides, and ceramides (Liu et al., 2022).

Recently, the investigations on lipid metabolites using mass spectrometry techniques such as mass spectrometry imaging (MSI) (Zhang et al., 2024), magnetic resonance spectroscopy (MRS) (Kantarci et al., 2007), direct infusion mass spectrometry(DIMS) (González-Domínguez et al., 2015a), Gas Chromatograph-Mass Spectrometer (GC-MS) (Wang et al., 2023), UPLC-Q/TOF-MS (Zhang et al., 2020), integration of GC-MS with UHPLC-MS(33)and others have primarily focused on untargeted lipidomics approaches (Strnad et al., 2019). However, to obtain a more precise conclusion that validates the results of untargeted lipid analysis, it is essential to conduct a subsequent validation stage employing a sensitive and accurate targeted method as our research method after untargeted lipid analyzing (González-Domínguez et al., 2014d). Our findings are only partially in line with the results of the existing literature on the lipidome associated with AD, perhaps partly due to the limitation of merely employing an untargeted approach in previous research (González-Domínguez et al., 2014a,b).

In recent years, the studies of lipids have been limited to nontargeted relative quantitative studies of serum, cerebrospinal fluid (CSF) or cerebral homogenate from the AD animal model. Quantitative investigations on hippocampal lipids remain necessary to be conducted (Zhang et al., 2020; Zhang et al., 2022; Liu et al., 2022; Proitsi et al., 2017).

Brain lipid homeostasis is strongly affected by hormonal condition from previous studies (Morselli et al., 2018; Marin and Diaz, 2018; Díaz et al., 2016; Herrera et al., 2019; Perez et al., 2010). Based on this conclusion, it can be inferred that the higher levels of estradiol in female mice result in a less severe degree of lipid disorder in their brain tissue compared to age-matched male mice. Therefore, all experimental animals in this study were male. If future research focuses on the hippocampal lipidomics of female AD mice, it may be necessary to increase the experimental age of female mice to achieve a comparable degree of lipid disorder.

Transgenic mouse models, such as APP/PS1 mice, are extensively utilized to investigate the pathological changes and metabolic disturbances associated with AD. APP/PS1 mice exhibit early pathological alterations as early as 4 months of age. By 6 months, AD mice display mild lipid modifications in the hippocampus. In the initial stages of AD, damage induced by factors like lipid metabolism disorders predominantly affects the hippocampus and cortical tissues. At 7 months, APP/PS1 mice already demonstrate cognitive deficits and lipid abnormalities. Given that circulating estradiol levels significantly influence lipid metabolism, this study exclusively employs male mice to avoid potential confounding effects from estrogen. The experimental timeline spans from 3.5 months, when initial brain pathological changes occur without evident cognitive alterations, to 7.5 months, when memory impairments and lipid-related damages become more pronounced. Despite these findings, current research on lipids in the hippocampus of AD mice predominantly relies on non-targeted lipidomics analyses, while quantitative targeted studies remain underexplored and warrant further investigation.

Lipids play pivotal roles in various biological processes, including the maintaining cellular structure such as membrane, membrane function, energy storage, signal transduction, gene expression regulation, and metabolic processes (Brügger, 2014; Sastry, 1985). Lipids are broadly classified into categories including fatty acyls (FA), glycerolipids (GL), glycerophospholipids (GP), sphingolipids (SP), sterol lipids (ST), prenol lipids (PR), polyketides (PK), and glycolipids, each with multiple subclasses. Over half of the human brain’s dry weight is lipid, consisting predominantly of phospholipids (50%), glycolipids (40%), and cholesterol (10%), with minor amounts of triglycerides and cholesterol esters (Kim and Song, 2024). Lipids are integral to neuronal membranes, and maintaining lipid homeostasis is crucial for preserving synaptic function (Zhang et al., 2020). Membrane lipid dysregulation contributes to AD pathogenesis, providing new avenues for understanding its etiology (Mielke and Lyketsos, 2006). Alterations in key membrane lipids and their metabolites correlate with AD onset and progression (Ariga, 2017). AD-like disorders in mouse models may involve multiple metabolic pathways of lipids, including disrupted membrane lipids and abnormal fatty acid compositions in phospholipids and sphingomyelins across various brain regions (González-Domínguez et al., 2014d).

First and foremost, we analyzed the functional roles of several pivotal lipids, such as Glycerophospholipids (GL), Sphingolipids (SP), CHOL in Sterol Lipids (ST), Glycolipids, and others. These lipids serve as primary constituents within neuronal membranes. The localization of these lipids is predominantly observed in neuronal membranes and myelin (Wang et al., 2021). Neuronal membranes contain hormone receptors, antigen-binding regions, and cell recognition sites, highlighting their crucial function in hormone signaling, immune reaction, and cellular communication.

Glycerophospholipids, essential phospholipid bilayer constituents, are involved in metabolism and signaling (Castro-Gómez et al., 2015). Key subclasses include phosphatidylcholine (PC), phosphatidylethanolamine (PE), and phosphatidylserine (PS). The nervous system is particularly rich in phospholipids, and changes in their composition may reflect or contribute to neurological dysfunction. Glycerophosphorylcholine is a metabolite of phosphatidylcholine, indicating an augmented decomposition and metabolism of phosphatidylcholine in individuals with AD (Zhang et al., 2020). Glycerophospholipids have been linked to amyloid deposition (Whiley et al., 2014), and aberrant phospholipid metabolism was found in AD models (Zhang et al., 2020). Aβ accumulation and related inflammation can enhance PLA2 activity, further degrading phosphatidylcholine and linking glycerophospholipid dysregulation to Aβ deposition (Zhang et al., 2020; Adibhatla et al., 2006). Elevated PC levels, a source of glycerophosphorylcholine, have been detected in CSF from AD patients and plasma from AD animals. These elevations correlate with abnormal Aβ1-42 levels and are associated with cognitive decline (Toledo et al., 2017; Liu et al., 2022; Huo et al., 2020). Our findings suggested that PC(16:0_20:3 + AcO) and PC(18:1_16:1 + AcO) might serve as characteristic markers, thus playing significant roles in AD mice. Connecting these findings with our previous studies indicates a possible link between increased levels of Aβ and the increase of PC(16:0_20:3 + AcO) and PC(18:1_16:1 + AcO). Previous studies have reported significant elevations in glycerophosphorylcholine and phosphorylcholine levels in the hippocampal portion from 6-month-aged AD mice, consistent with the findings presented in our research (González-Domínguez et al., 2014d).

Glycerophospholipids, including phosphatidylcholine (PC), play a pivotal role in lipid metabolism. Abnormal PC metabolism has been linked to amyloid deposition and cognitive decline in AD models. Elevated levels of PC, which serves as the precursor for glycerophosphocholine, have been observed in the cerebrospinal fluid of AD patients and the plasma of AD animal models. The metabolic disturbances of PC(16:0_20:3 + AcO) and PC(18:1_16:1 + AcO) identified in the hippocampal tissue of AD mice in this study indicate that acetylated phosphatidylcholine may contribute to hippocampal tissue damage during disease progression.

The enrichment of PE in mitochondrial membranes endows it with the regulatory function over autophagy. Changes in PE levels have forecasted the progression from mild cognitive impairment to AD (Li et al., 2023), and reduced phosphoethanolamine (the precursor to PE) has been noted in postmortem AD brains (Klunk et al., 1996), further supporting the presence of a membrane defect in AD. Furthermore, previous studies have observed a substantial decrease in different PEs of phosphatidylethanolamine in various brain regions of AD mice (González-Domínguez et al., 2015a,b; Li et al., 2023). In our investigation, a decline was also noted in the majority of the measured PE species in the hippocampus of 7.5-month-aged AD mice, including PE(P-16:0_16:1), PE(P-18:0_16:1), PE(P-18:1_18:0), PE(P-18:1_18:2), PE(P-18:1_20:3), and PE(P-18:0_20:3).

The phenomenon of decreased PEs has also been elucidated in previous literature on AD patients and AD mice (Han et al., 2001; Chan et al., 2012). In addition, numerous studies, including our research on the hippocampus of APP/PS1 mice (Han et al., 2017; Liu et al., 2022; González-Domínguez et al., 2014b; Igarashi et al., 2011), reported reduced levels of several PEs, but the kind of decreased PEs was not the same as our data in the research. These PE reductions may result from increased PLA2 activity in AD brains (Frisardi et al., 2011). Notably, the lipid composition of PEs differed from that observed in serum and brain tissue from AD mice (Zhang et al., 2020). Decreased PE levels may contribute to membrane disruption and impaired neuronal function, potentially underpinning memory deficits and serving as biomarkers of injury severity in AD. Conversely, elevated levels of certain PEs (PE(O-16:0_22:4), PE(P-16:0_20:4)) and PCs (PC(16:0_20:3 + AcO), PC(18:1_16:1 + AcO)) suggest active membrane remodeling in the APP/PS1 mice’s hippocampus, consistent with prior findings (González-Domínguez et al., 2015a).

Sphingolipids (SPs), including sphingomyelins, ceramides, and sphingosines, influence membrane properties and signaling. They can interconvert via well-characterized metabolic pathways (Merrill, 2011; Chua et al., 2023). Sphingomyelin, as a crucial lipid constituent of the myelin sheath, is pivotal in facilitating the conduction of electrical impulses along the axons. Within the central nervous system (CNS), sphingomyelin may stimulate amyloid precursor protein and neuronal activity, therefore affecting the development of AD (Qiang et al., 2024). The global downregulation of SPs, irrespective of their class, was found to be in relation to cardiovascular disease, which is an exact risk factor for dementia, reported in the previous study (Chua et al., 2023; Qiang et al., 2024). SPs serve as integral components of the biological membrane structure. Through interactions with other constituents, SPs facilitate the formation of membrane lipid rafts. Disrupted sphingomyelin levels in APP/PS1 mice reflect abnormal membrane homeostasis and lipid raft function (Fabelo et al., 2012).

The well-documented regulatory roles of sphingomyelins (SMs), ceramides (Cers), and other sphingolipids span a broad spectrum of biological course, such as cell growth, adhesion, differentiation, and apoptosis (Shen S. et al., 2017). Research links elevated sphingomyelin (SM) levels to AD severity, including cognitive decline and brain atrophy (Toledo et al., 2017; Liu et al., 2022; Varma et al., 2018). However, postmortem analyses have reported overall SM reductions in AD brains (Cutler et al., 2004). The involvement of ceramide in regulating neuronal survival and apoptosis, as well as its responsibility in pathological processes of AD, is evident within the intricate network in the nervous system (Czubowicz et al., 2019).

However, our report revealed only a decrease in SM(24:0), Cer(d18:1/18:1), Cer(d18:1/16:1) and Cer(d18:1/24:1)levels was explicitly shown in the hippocampal tissue of 7.5-month-old AD mice, whereas previous research reported a decline in SM levels only at 2 and 3 months old and no change at 7 months old (Zhang et al., 2020). The observed reduction in CER levels did not align with the findings of the previous study when examining plasma samples from 9-month-old AD mice (Zhang et al., 2020; Liu et al., 2022). Accordingly, I assumed that the release of SPs from the destroyed cellular membrane in the hippocampus of AD may contribute to its elevation in bloodstream through cerebral vessels. Consequently, this led to an elevation in the levels of SPs within both the bloodstream and CSF of patients diagnosed with AD (Toledo et al., 2017; Kosicek et al., 2012). However, further verification is required to confirm the assumption.

Ceramide serves as the core node of the sphingolipid metabolism, encompassing hexose ceramide (HexCer), lactose ceramide (LacCer), sphingosine (Sph), dihydrosphingosine (DhSph), and dihydroceramide (DhSph). The levels of HexCer exhibit dynamic changes across various physiological, pathological, and disease conditions. Research has shown that the levels of HexCer undergo changes during functional decline of brain. In our investigation, the concentrations of the majority of HexCers in the hippocampus of 7.5-month-old AD mice exhibited a substantial reduction. In essence, the cerebral function depends upon the assistance of HexCer and SM in nerve sheaths, as these chemical substances form the crucial components of myelin sheaths within the central nervous system.

Myelin sheaths rapidly transmit information about bodily activities to the brain via neurotransmitters and facilitate normal cognitive and memory development in humans. Membrane disruption in AD has usually been attributed to the overactivation of phospholipases, particularly phospholipase A2, degrading sphingolipids and generating neurodegenerative second messengers (Farooqui et al., 2004). So, reducing SM, Cer, and HexCer levels in the hippocampal tissue may contribute to cognitive impairment in 7.5-month-old AD mice. Alternatively, the decreased SPs, a vital cellular membrane constituent, can potentially to disrupt neuronal structure and impair cognitive function. Moreover, it may also compromise microglial functionality in the hippocampus, diminishing their ability to phagocytose toxic Aβ and consequently impacting memory. Based on our research findings, there appeared to be a positive correlation various types of Cer and HexCer and potential synergistic interactions between them. Consequently, these results rise whether positive feedback or mutually reinforcing promotion exists within different species of Cer, HexCer, or even between them. Dysregulated membrane integrity in APP/PS1 mice is strongly influenced by sphingolipid metabolism, as evidenced by previous research indicating reduced sphingomyelins and sphingoid bases. Disrupted sphingomyelin metabolism is proposed as a critical factor in neuronal dysfunction and degeneration in AD (Fukuhara et al., 2013).

Lipid rafts, cholesterol-enriched micro domains on the membrane, facilitate Aβ peptides generation and oligomerization. An abundance of compelling evidence has unequivocally associated lipid rafts with neurodegenerative diseases, including AD (Mesa-Herrera et al., 2019). We therefore assumed that dysregulated sphingolipids, glycerophospholipids, and cholesterol metabolism disrupts lipid raft homeostasis in the hippocampus of AD mice, leading to aberrant Aβ expression. Given that lipid rafts are key sites for protein docking, signal transduction, and substrate transport, their perturbation may underlie the observed memory deficits in AD mice. Interestingly, while some studies report decreased cholesterol levels in AD transgenic mouse brains (González-Domínguez et al., 2014d; Fabelo et al., 2012), our findings in the hippocampus differ, showing an opposite trend. Cholesterol and related sterols within lipid rafts are crucial lipids implicated in the pathogenesis of hyperlipidemia and atherosclerosis, leading to hypertension and cardiovascular diseases. Recent research, including ours, show increased cholesterol in AD mouse brains, reinforcing evidence that cholesterol dysregulation is integral to AD pathophysiology (Zhang et al., 2020; Di Paolo and Kim, 2011). The association between cholesterol levels and AD may stem from its involvement in neuronal pathways that foster Aβ formation (Ahmed et al., 2024). A recent study further suggested that cholesterol can catalyze Aβ1-42 aggregation, highlighting its central role in AD pathogenesis (Habchi et al., 2018).

The decreased sphingomyelin in the hippocampal tissue of APP/PS1 mice may affect cholesterol metabolism through various mechanisms, such as attenuating the conversion of cholesterol to bile acids or cholesterol ester, inhibiting the activity of HMG-CoA, promoting cholesterol biosynthesis. Accordingly, individuals with vascular risk factors, including hypercholesterolemia, have a higher incidence of both vascular dementia and AD (Breteler, 2000). These conditions reduce cerebral blood flow and may precipitate neuronal loss. However, the conclusions drawn from the previous research were inconsistent due to variations in processing, extracting and detecting methods of lipid metabolites in AD mice’s hippocampus (Söderberg et al., 1990; Svennerholm et al., 1994). Total cholesterol (TC) in the hippocampus of mice refers to the cumulative cholesterol content within the hippocampal tissue. It can be further categorized into esterifies cholesterol (CE) and free cholesterol (FC), with CE constituting approximately 60 to 70%, while FC accounts for around 30 to 40%. In our study, CE(22:6) and CE(22:4)—associated with atherosclerosis—were elevated in the hippocampus of APP/PS1 mice. This increase may promote Aβ1-42 aggregation, hinder its vascular clearance, and ultimately slow cerebral blood flow and compromise blood vessel integrity. This is the important study to demonstrate the elevated CE(22:6) and CE(22:4) levels in the hippocampus of 7.5-month-old APP/PS1 mice. These findings provided new evidence to the ongoing debate regarding cholesterol’s role in AD pathogenesis.

Linoleic acid (LA, FA(18:2)) is an essential polyunsaturated fatty acid that serves as a precursor to eicosanoids, regulating vascular tone and inflammatory responses across various brain regions (Jantzen et al., 2024). The compound is a crucial structural constituent of cellular membranes and is the precursor to the ω6 PUFA family (Qiang et al., 2024; Whelan and Fritsche, 2013). It can undergo esterification to generate phospholipids, which constitute the principal constituents of cellular membranes (Whelan and Fritsche, 2013). Its remarkable biological significance lies in its ability to fulfill its own functions and its capacity to generate various unsaturated fatty acids, such as DHA. DHA supports brain structure and function by maintaining membrane fluidity, improving mitochondrial function, reducing neuroinflammation, and facilitating glucose uptake (Sinclair, 2019; Pifferi et al., 2015). The administration of docosahexaenoic acid (DHA) has been shown to enhance cerebral energy metabolism in APP/PS1 mice, thereby attenuating neuronal apoptosis and suppressing amyloid-beta (Aβ) expression, as demonstrated by previous research (Wang et al., 2023). Multiple studies linked elevated plasma LA to a lower risk of dementia (Qiang et al., 2024; He et al., 2023; Morris et al., 2003). Accordingly, reduced LA levels may increase dementia risk. The regulatory roles of LA in microglial function and neuroinflammation have been extensively characterized, as demonstrated by the decrease of NO formation in immune-stimulated BV-2 microglia and the down regulation of iNOS expression in AD mice (Lowry et al., 2020).

Conversely, we concluded that a reduction in LA might be relevant to an increased risk of AD development. However, no existing literature reported on LA in the hippocampal tissue of 7.5-month-old AD mice. We report reduced LA in this region and propose mechanistic explanations for this finding. LA supports neuronal mitochondrial bioenergetics. Its reduction may promote mitochondrial dysfunction, accelerating the formation of Aβ plaque and neurofibrillary tangle formation and impairing cognitive functions.

We reported, for the first time, decreased FA(18:2) in the AD hippocampus potentially explained why Aβ accumulated and memory impaired in these mice, as evidenced by our previous experiments. The efficacy of linoleic acid (FA(18:2)) in reducing blood cholesterol, LDL-apoB, and increasing the HDL-C level has been extensively investigated, demonstrating its potential as a valuable intervention (Kim and Song, 2024). The impaired glucose metabolites and memory in AD mice may be attributed to the reduced levels of linoleic acid in the hippocampus in our previous research (Shen L. et al., 2017). So, the decreased levels of linoleic acid (FA(18:2)) as the substrate of Lecithin Cholesterol Acyl Transferase (LCAT) in hippocampal tissue can reduce its ability to combine with cholesterol and form esters. This impairment can elevate cholesterol levels in the AD hippocampus, depositing on cerebral vessel walls (Kim and Song, 2024). Consequently, this can decrease vascular elasticity and promote the development of cerebral atherosclerosis. Reduced LA may elevate cholesterol, LDL-C, and other lipids in the brain, hindering their metabolism and promoting vascular sclerosis. This vascular impairment likely impedes Aβ clearance from the hippocampus into the circulation of AD mice (Kim and Song, 2024). In the previous research, linoleic acid could accelerate blood circulation (Jantzen et al., 2024). A reduction in the level of linoleic acid can decelerate blood flow, decrease metabolism, and contribute to the accumulation of excessive lipids, including CHOL and LDL-C in cerebrovascular, thereby further promoting the formation of atherosclerosis in cerebral arteries, reducing brain energy supply and thus diminishing cognitive levels in AD (Kim and Song, 2024). As an unsaturated fatty acid, linoleic acid stimulates brain cell development and augments their activity. It also converts arachidonic acid into a precursor for neurotransmitters, exerting a promotive effect on neural function. A decline in FA (18:2) within hippocampal tissue will result in reduced brain cell activity and impaired memory function within the nervous system of AD mice. The crucial point lies in the antioxidant and anti-neuroinframmatory properties exhibited by linoleic acid (Jantzen et al., 2024). A reduction in hippocampal tissue levels of linoleic acid results in a decline in its capacity to counteract the damage caused by increased free radicals and neuroinframmation due to Aβ accumulation. Consequently, this reduction of LC may expedite the development of cerebral arteriosclerosis, impede blood circulation, elevate blood lipids (including triglycerides and cholesterol), decrease metabolism, and ultimately impair cognitive function (Kim and Song, 2024). Indeed, reduced LA has been detected in the CSF of AD patients (Turri et al., 2023).

Glycerolipids (GL) encompass monoglycerides (MG), diglycerides (DG), and triglycerides (TG), with the most important being triglyceride. Although prior literature reported diglycerides (DG) changes in AD mouse brains, we found no significant DG alterations in the hippocampus. Instead, we observed increased levels of multiple triglycerides (TGs) within the hippocampal tissue of APP/PS1 mice. Elevated TGs’ levels suggest hyperlipidemia, a key vascular risk factor in AD pathogenesis. Triglycerides’ accumulation promotes atherosclerosis and endothelial dysfunction, hindering the clearance of Aβ42 and Aβ40 through cerebral circulation. Previous studies show increased serum TG in both AD patients and mice, correlating with disease severity (González-Domínguez et al., 2014c; Tajima et al., 2013). In relation to glycerolipids, the concentration of triglycerides (TGs) were notably elevated in APP/PS1 mice, and our findings are in agreement with prior studies reporting increased TG levels in the serum of AD patients (Liu et al., 2022; Berezhnoy et al., 2022). The hippocampus was significantly affected that TGs were obviously elevated though it occupied a relatively small volume in the entire brain. Based on prior studies, a panel of biomarkers could represent the pathophysiological condition of AD mice at the age of 7 months (Laske et al., 2011).

However, in our previous study, glucose metabolites showed significant differences compared to WT mice using GS-MS analysis, and there was relative mild hippocampal lipid damage in AD mice. We thus delayed deadline of experiment to identify a more suitable time point—7.5 months—to explore lipid-AD relationships and discover potential AD lipid biomarkers. In the correlation analysis conducted in our research, TG (54:4)_FA(20:4) exhibited a significantly positive correlation with other components of TAGs, including TG (54:6)_FA(22:6) (r = 0.801) and TG(56:4)_FA(20:4) (r = 0.898). Based on these findings, it is plausible to suggest that there may be mutual promotion among TGs, which has not been previously reported in recent research.

In the second part of the discussion, atherosclerosis-related lipid metabolites—such as CEs, TGs, and FAs—offered insights into neurochemical alterations underlying AD. This offered a thorough insight into the hippocampal condition associated with cognitive and memory functions, thereby reflecting intricate interactions among genes, proteins, and environmental factors (Lindon et al., 2004). These unbalanced lipids, as vascular risk factors, were linked to elevated AD prevalence by reducing cerebral blood flow and contributing to neuronal loss.

Glycolipids, which are lipids covalently bonded to carbohydrates through glycoside linkages, serve as crucial recognition sites for cell–cell interactions in the body. These interactions between cell surface markers play a fundamental role in maintaining membrane stability, facilitating cell recognition, and initiating cellular responses that contribute to regulatory processes, growth regulation, apoptosis induction, and immune responses. Presenting on all eukaryotic cell surfaces, glycolipids project from the phospholipids’ bilayer into the extracellular space. Thus, when membrane integrity is destroyed—due to decreased HexCers, PEs, and other key lipids—monogalactosyldiacylglycerols (MGDGs) also diminish at the membrane surface.

MGDG, a galactose-based glyceroglycolipid, is a key membrane constituent and signaling molecule. It also exhibits COX-mediated anti-inflammatory effects (Guo and Wang, 2022). Previous studies have reported that MGDG can exhibit anti-inflammatory effects by inhibiting P38 phosphorylation; However, the direct target of MGDG remains unclear (Liu et al., 2016). In addition to exerting anti-inflammatory effects, glycolipid mono galactosyl diglycerides (MGDGs) also exhibit diverse biological activities, including scavenging oxygen free radicals and antioxidant properties such as suppressing NO synthesis by down-regulating inducible NO synthase (Guo and Wang, 2022; Banskota et al., 2013).

The third part of the discussion focused on lipids that were linked to antioxidant and anti-inflammatory effects. We found that most MGDGs were significantly reduced in the AD hippocampus. The decrease likely impaired the tissue’s ability to counteract inflammation and oxidative stress. Thus, enhancing MGDG levels might represent a novel therapeutic strategy to strengthen hippocampal defenses against Aβ-induced inflammation and ameliorate memory deficits in AD.

Acetylated phosphatidylcholine such as PC (16:0_16:1 + AcO) and so on, characterized by specific acyl chain modifications in its molecular structure, is a modified form of phosphatidylcholine (PC). This compound may represent an oxidized or acetylated variant of phosphatidylcholine, and its structural features are closely associated with lipid peroxidation damage, neuroinflammation, and Aβ-induced toxicity observed in Alzheimer’s disease (AD). Elevated oxidized or acetylated phospholipids levels have been detected in AD patients’ serum, potentially as indicators of lipid peroxidation-related damage (Dong and Yong, 2022). The research demonstrating elevated lipid peroxidation in the hippocampus of AD mice may further reinforce the role of oxidative damage in hippocampal tissue pathology.

In summary, the reduced levels of phosphatidylethanolamine (PE) in the hippocampus of AD mice are closely associated with AD progression, potentially impairing neuronal function via disrupted autophagy regulation and membrane structural damage. Dysregulated sphingolipid metabolism, characterized by decreased sphingomyelin, ceramide, and hexosylceramide, further exacerbates cognitive deficits and neuroinflammation in AD mice. Abnormal cholesterol metabolism in the hippocampus, particularly elevated esterified cholesterol (CE), may promote Aβ aggregation and vascular dysfunction, thereby intensifying pathological damage in AD mice. The decline in linoleic acid (LA) levels within the hippocampus could lead to mitochondrial dysfunction and heightened neuroinflammation, disrupting brain energy metabolism and accelerating AD progression. Moreover, increased triglyceride (TG) levels in the hippocampus, as a marker of hyperlipidemia, may impede Aβ clearance through atherosclerotic mechanisms, serving as a critical vascular risk factor for AD. The reduction in glycolipids such as monogalactosyldiacylglycerol (MGDG) weakens anti-inflammatory and antioxidant capacities in the hippocampus of AD mice, highlighting their potential therapeutic significance.

Acetylated phosphatidylcholine is a variant of phosphatidylcholine modified by acyl chains. Its oxidized or acetylated structure is closely associated with lipid peroxidation damage, neuroinflammation, and Aβ-mediated toxicity in Alzheimer’s disease (AD). The levels of these modified lipids are elevated in the hippocampus of AD mice, potentially reflecting lipid peroxidation-related pathological damage.

## Conclusion

This study employed a targeted UHPLC-MS/MS method to identify hippocampal lipid biomarkers in 7.5-month-old AD (APP/PS1) mice. The alterations in the lipids including sphingolipids, glycerophospholipids, sterol lipids, and glycolipids, were closely associated with membrane damage observed in the hippocampus of 7.5-month-old AD mice. Furthermore, the altered lipids such as glycerides, sterol lipids, and free fatty acids, were linked to the upward vascular risk, whereas those decreased lipids such as glycolipids were associated with reduced antioxidant and anti-inflammatory capacities of the hippocampus in early-onset AD mice. These results suggest that lipid metabolite disorders may underlie the mechanisms responsible for Aβ accumulation, impaired glucose metabolism, and cognitive/memory deficits observed in early-onset AD mice. Based on our research, we conclude that strategic exogenous supplementation of key lipids in cellular membranes, combined with modulation of lipid levels to mitigate atherosclerosis, inflammation, and oxidative stress, holds promise as a future therapeutic strategy for early-onset Alzheimer’s disease.

## Data Availability

The original contributions presented in the study are included in the article/Supplementary material, further inquiries can be directed to the corresponding authors.

## References

[ref1] Adibhatla R. M. Hatcher J. F. Larsen E. C. Chen X. Sun D. Tsao F. H. (2006). CDP-choline significantly restores phosphatidylcholine levels by differentially affecting phospholipase A2 and CTP: phosphocholine cytidylyltransferase after stroke. J. Biol. Chem. 281, 6718–6725. doi: 10.1074/jbc.M512112200, PMID: 16380371

[ref2] Ahmed H. Wang Y. Griffiths W. J. Levey A. I. Pikuleva I. Liang S. H.. (2024). Brain cholesterol and Alzheimer's disease: challenges and opportunities in probe and drug development. Brain 147, 1622–1635. doi: 10.1093/brain/awae028, PMID: 38301270 PMC11068113

[ref3] Alseekh S. Aharoni A. Brotman Y. Contrepois K. D’Auria J. Ewald J.. (2021). Mass spectrometry-based metabolomics: a guide for annotation, quantification and best reporting practices. Nat. Methods 18, 747–756. doi: 10.1038/s41592-021-01197-1, PMID: 34239102 PMC8592384

[ref4] Alzheimer A. Stelzmann R. A. Schnitzlein H. N. Murtagh F. R. (1995). An English translation of Alzheimer's 1907 paper, "Uber eine eigenartige Erkankung der Hirnrinde". Clin. Anat. 8, 429–431. doi: 10.1002/ca.980080612, PMID: 8713166

[ref5] Ariga T. (2017). The pathogenic role of ganglioside metabolism in Alzheimer's disease-cholinergic neuron-specific gangliosides and neurogenesis. Mol. Neurobiol. 54, 623–638. doi: 10.1007/s12035-015-9641-0, PMID: 26748510

[ref6] Arvanitakis Z. Capuano A. W. Leurgans S. E. Bennett D. A. Schneider J. A. (2016). Relation of cerebral vessel disease to Alzheimer's disease dementia and cognitive function in elderly people: a cross-sectional study. Lancet Neurol. 15, 934–943. doi: 10.1016/s1474-4422(16)30029-1, PMID: 27312738 PMC4969105

[ref7] Attems J. Jellinger K. A. (2014). The overlap between vascular disease and Alzheimer's disease--lessons from pathology. BMC Med. 12:206. doi: 10.1186/s12916-014-0206-2, PMID: 25385447 PMC4226890

[ref8] Banskota A. H. Gallant P. Stefanova R. Melanson R. O'Leary S. J. (2013). Monogalactosyldiacylglycerols, potent nitric oxide inhibitors from the marine microalga Tetraselmis chui. Nat. Prod. Res. 27, 1084–1090. doi: 10.1080/14786419.2012.717285, PMID: 22973805

[ref9] Berezhnoy G. Laske C. Trautwein C. (2022). Quantitative NMR-based lipoprotein analysis identifies elevated HDL-4 and triglycerides in the serum of Alzheimer's disease patients. Int. J. Mol. Sci. 23:23. doi: 10.3390/ijms232012472, PMID: 36293327 PMC9604278

[ref10] Braak H. Braak E. (1991). Neuropathological stageing of Alzheimer-related changes. Acta Neuropathol. 82, 239–259. doi: 10.1007/bf00308809, PMID: 1759558

[ref11] Breteler M. M. (2000). Vascular risk factors for Alzheimer's disease: an epidemiologic perspective. Neurobiol. Aging 21, 153–160. doi: 10.1016/s0197-4580(99)00110-4, PMID: 10867200

[ref12] Brügger B. (2014). Lipidomics: analysis of the lipid composition of cells and subcellular organelles by electrospray ionization mass spectrometry. Annu. Rev. Biochem. 83, 79–98. doi: 10.1146/annurev-biochem-060713-035324, PMID: 24606142

[ref13] Button E. B. Boyce G. K. Wilkinson A. Stukas S. Hayat A. Fan J.. (2019). ApoA-I deficiency increases cortical amyloid deposition, cerebral amyloid angiopathy, cortical and hippocampal astrogliosis, and amyloid-associated astrocyte reactivity in APP/PS1 mice. Alzheimers Res. Ther. 11:44. doi: 10.1186/s13195-019-0497-9, PMID: 31084613 PMC6515644

[ref14] Calsolaro V. Antognoli R. Okoye C. Monzani F. (2019). The use of antipsychotic drugs for treating behavioral symptoms in Alzheimer's disease. Front. Pharmacol. 10:1465. doi: 10.3389/fphar.2019.01465, PMID: 31920655 PMC6915160

[ref15] Castro-Gómez P. Garcia-Serrano A. Visioli F. Fontecha J. (2015). Relevance of dietary glycerophospholipids and sphingolipids to human health. Prostaglandins Leukot. Essent. Fatty Acids 101, 41–51. doi: 10.1016/j.plefa.2015.07.004, PMID: 26242691

[ref16] Chan R. B. Oliveira T. G. Cortes E. P. Honig L. S. Duff K. E. Small S. A.. (2012). Comparative lipidomic analysis of mouse and human brain with Alzheimer disease. J. Biol. Chem. 287, 2678–2688. doi: 10.1074/jbc.M111.274142, PMID: 22134919 PMC3268426

[ref17] Chua X. Y. Torta F. Chong J. R. Venketasubramanian N. Hilal S. Wenk M. R.. (2023). Lipidomics profiling reveals distinct patterns of plasma sphingolipid alterations in Alzheimer's disease and vascular dementia. Alzheimers Res. Ther. 15:214. doi: 10.1186/s13195-023-01359-7, PMID: 38087395 PMC10714620

[ref18] Cutler R. G. Kelly J. Storie K. Pedersen W. A. Tammara A. Hatanpaa K.. (2004). Involvement of oxidative stress-induced abnormalities in ceramide and cholesterol metabolism in brain aging and Alzheimer's disease. Proc. Natl. Acad. Sci. USA 101, 2070–2075. doi: 10.1073/pnas.0305799101, PMID: 14970312 PMC357053

[ref19] Czubowicz K. Jęśko H. Wencel P. Lukiw W. J. Strosznajder R. P. (2019). The role of ceramide and Sphingosine-1-phosphate in Alzheimer's disease and other neurodegenerative disorders. Mol. Neurobiol. 56, 5436–5455. doi: 10.1007/s12035-018-1448-3, PMID: 30612333 PMC6614129

[ref20] de Veij Mestdagh C. F. Koopmans F. Breiter J. C. Timmerman J. A. Vogelaar P. C. Krenning G.. (2022). The hibernation-derived compound SUL-138 shifts the mitochondrial proteome towards fatty acid metabolism and prevents cognitive decline and amyloid plaque formation in an Alzheimer's disease mouse model. Alzheimers Res. Ther. 14:183. doi: 10.1186/s13195-022-01127-z, PMID: 36482297 PMC9733344

[ref21] Di Paolo G. Kim T. W. (2011). Linking lipids to Alzheimer's disease: cholesterol and beyond. Nat. Rev. Neurosci. 12, 284–296. doi: 10.1038/nrn3012, PMID: 21448224 PMC3321383

[ref22] Díaz M. Fabelo N. Casañas-Sánchez V. Marin R. Gómez T. Quinto-Alemany D.. (2016). Hippocampal lipid homeostasis in APP/PS1 mice is modulated by a complex interplay between dietary DHA and estrogens: relevance for Alzheimer's disease. J. Alzheimers Dis. 49, 459–481. doi: 10.3233/jad-150470, PMID: 26519437

[ref23] Dong Y. Yong V. W. (2022). Oxidized phospholipids as novel mediators of neurodegeneration. Trends Neurosci. 45, 419–429. doi: 10.1016/j.tins.2022.03.002, PMID: 35393134

[ref24] Dunn W. B. Broadhurst D. Begley P. Zelena E. Francis-McIntyre S. Anderson N.. (2011). Procedures for large-scale metabolic profiling of serum and plasma using gas chromatography and liquid chromatography coupled to mass spectrometry. Nat. Protoc. 6, 1060–1083. doi: 10.1038/nprot.2011.335, PMID: 21720319

[ref25] Fabelo N. Martín V. Marín R. Santpere G. Aso E. Ferrer I.. (2012). Evidence for premature lipid raft aging in APP/PS1 double-transgenic mice, a model of familial Alzheimer disease. J. Neuropathol. Exp. Neurol. 71, 868–881. doi: 10.1097/NEN.0b013e31826be03c, PMID: 22975585

[ref26] Fan X. Liu B. Zhou J. Gu X. Zhou Y. Yang Y.. (2021). High-fat diet alleviates Neuroinflammation and metabolic disorders of APP/PS1 mice and the intervention with Chinese medicine. Front. Aging Neurosci. 13:658376. doi: 10.3389/fnagi.2021.658376, PMID: 34168550 PMC8217439

[ref27] Farooqui A. A. Liss L. Horrocks L. A. (1988). Neurochemical aspects of Alzheimer's disease: involvement of membrane phospholipids. Metab. Brain Dis. 3, 19–35. doi: 10.1007/bf01001351, PMID: 3062351

[ref28] Farooqui A. A. Ong W. Y. Horrocks L. A. (2004). Biochemical aspects of neurodegeneration in human brain: involvement of neural membrane phospholipids and phospholipases A2. Neurochem. Res. 29, 1961–1977. doi: 10.1007/s11064-004-6871-3, PMID: 15662832

[ref29] Foley P. (2010). Lipids in Alzheimer's disease: a century-old story. Biochim. Biophys. Acta 1801, 750–753. doi: 10.1016/j.bbalip.2010.05.004, PMID: 20471492

[ref30] Frisardi V. Panza F. Seripa D. Farooqui T. Farooqui A. A. (2011). Glycerophospholipids and glycerophospholipid-derived lipid mediators: a complex meshwork in Alzheimer's disease pathology. Prog. Lipid Res. 50, 313–330. doi: 10.1016/j.plipres.2011.06.001, PMID: 21703303

[ref31] Fukuhara K. Ohno A. Ota Y. Senoo Y. Maekawa K. Okuda H.. (2013). NMR-based metabolomics of urine in a mouse model of Alzheimer's disease: identification of oxidative stress biomarkers. J. Clin. Biochem. Nutr. 52, 133–138. doi: 10.3164/jcbn.12-118, PMID: 23526113 PMC3593130

[ref32] Gengler S. Hamilton A. Hölscher C. (2010). Synaptic plasticity in the hippocampus of a APP/PS1 mouse model of Alzheimer's disease is impaired in old but not young mice. PLoS One 5:e9764. doi: 10.1371/journal.pone.0009764, PMID: 20339537 PMC2842299

[ref33] González-Domínguez R. García-Barrera T. Gómez-Ariza J. L. (2014a). Combination of metabolomic and phospholipid-profiling approaches for the study of Alzheimer's disease. J. Proteome 104, 37–47. doi: 10.1016/j.jprot.2014.01.014, PMID: 24473279

[ref34] González-Domínguez R. García-Barrera T. Gómez-Ariza J. L. (2014b). Metabolomic study of lipids in serum for biomarker discovery in Alzheimer's disease using direct infusion mass spectrometry. J. Pharm. Biomed. Anal. 98, 321–326. doi: 10.1016/j.jpba.2014.05.023, PMID: 24992214

[ref35] González-Domínguez R. García-Barrera T. Gómez-Ariza J. L. (2014c). Using direct infusion mass spectrometry for serum metabolomics in Alzheimer's disease. Anal. Bioanal. Chem. 406, 7137–7148. doi: 10.1007/s00216-014-8102-3, PMID: 25230597

[ref36] González-Domínguez R. García-Barrera T. Vitorica J. Gómez-Ariza J. L. (2014d). Region-specific metabolic alterations in the brain of the APP/PS1 transgenic mice of Alzheimer's disease. Biochim. Biophys. Acta 1842, 2395–2402. doi: 10.1016/j.bbadis.2014.09.014, PMID: 25281826

[ref37] González-Domínguez R. García-Barrera T. Vitorica J. Gómez-Ariza J. L. (2015a). Metabolomic screening of regional brain alterations in the APP/PS1 transgenic model of Alzheimer's disease by direct infusion mass spectrometry. J. Pharm. Biomed. Anal. 102, 425–435. doi: 10.1016/j.jpba.2014.10.009, PMID: 25459942

[ref38] González-Domínguez R. García-Barrera T. Vitorica J. Gómez-Ariza J. L. (2015b). Deciphering metabolic abnormalities associated with Alzheimer's disease in the APP/PS1 mouse model using integrated metabolomic approaches. Biochimie 110, 119–128. doi: 10.1016/j.biochi.2015.01.005, PMID: 25597416

[ref39] Guo S. S. Wang Z. G. (2022). Glyceroglycolipids in marine algae: a review of their pharmacological activity. Front. Pharmacol. 13:1008797. doi: 10.3389/fphar.2022.1008797, PMID: 36339569 PMC9633857

[ref40] Habchi J. Chia S. Galvagnion C. Michaels T. C. T. Bellaiche M. M. J. Ruggeri F. S.. (2018). Cholesterol catalyses Aβ42 aggregation through a heterogeneous nucleation pathway in the presence of lipid membranes. Nat. Chem. 10, 673–683. doi: 10.1038/s41557-018-0031-x, PMID: 29736006

[ref41] Han X. Holtzman D. M. McKeel D. W. Jr. (2001). Plasmalogen deficiency in early Alzheimer's disease subjects and in animal models: molecular characterization using electrospray ionization mass spectrometry. J. Neurochem. 77, 1168–1180. doi: 10.1046/j.1471-4159.2001.00332.x, PMID: 11359882

[ref42] Han B. Wang J. H. Geng Y. Shen L. Wang H. L. Wang Y. Y.. (2017). Chronic stress contributes to cognitive dysfunction and hippocampal metabolic abnormalities in APP/PS1 mice. Cell. Physiol. Biochem. 41, 1766–1776. doi: 10.1159/000471869, PMID: 28365686

[ref43] He X. Huang Y. Li B. Gong C. X. Schuchman E. H. (2010). Deregulation of sphingolipid metabolism in Alzheimer's disease. Neurobiol. Aging 31, 398–408. doi: 10.1016/j.neurobiolaging.2008.05.010, PMID: 18547682 PMC2829762

[ref44] He Y. Huang S. Y. Wang H. F. Zhang W. Deng Y. T. Zhang Y. R.. (2023). Circulating polyunsaturated fatty acids, fish oil supplementation, and risk of incident dementia: a prospective cohort study of 440,750 participants. Geroscience 45, 1997–2009. doi: 10.1007/s11357-023-00778-6, PMID: 37046127 PMC10400523

[ref45] Heijboer A. C. Donga E. Voshol P. J. Dang Z. C. Havekes L. M. Romijn J. A.. (2005). Sixteen hours of fasting differentially affects hepatic and muscle insulin sensitivity in mice. J. Lipid Res. 46, 582–588. doi: 10.1194/jlr.M400440-JLR200, PMID: 15576835

[ref46] Herrera J. L. Ordoñez-Gutierrez L. Fabrias G. Casas J. Morales A. Hernandez G.. (2019). Ovarian hormone-dependent effects of dietary lipids on APP/PS1 mouse brain. Front. Aging Neurosci. 11:346. doi: 10.3389/fnagi.2019.00346, PMID: 31920626 PMC6930904

[ref47] Hill E. Goodwill A. M. Gorelik A. Szoeke C. (2019). Diet and biomarkers of Alzheimer's disease: a systematic review and meta-analysis. Neurobiol. Aging 76, 45–52. doi: 10.1016/j.neurobiolaging.2018.12.008, PMID: 30682676

[ref48] Huang Y. Guo B. Shi B. Gao Q. Zhou Q. (2018). Chinese herbal medicine Xueshuantong enhances cerebral blood flow and improves neural functions in Alzheimer's disease mice. J. Alzheimers Dis. 63, 1089–1107. doi: 10.3233/jad-170763, PMID: 29710701 PMC6004915

[ref49] Huo Z. Yu L. Yang J. Zhu Y. Bennett D. A. Zhao J. (2020). Brain and blood metabolome for Alzheimer's dementia: findings from a targeted metabolomics analysis. Neurobiol. Aging 86, 123–133. doi: 10.1016/j.neurobiolaging.2019.10.014, PMID: 31785839 PMC6995427

[ref50] Igarashi M. Ma K. Gao F. Kim H. W. Rapoport S. I. Rao J. S. (2011). Disturbed choline plasmalogen and phospholipid fatty acid concentrations in Alzheimer's disease prefrontal cortex. J. Alzheimers Dis. 24, 507–517. doi: 10.3233/jad-2011-101608, PMID: 21297269 PMC3175096

[ref51] Jankowsky J. L. Fadale D. J. Anderson J. Xu G. M. Gonzales V. Jenkins N. A.. (2004). Mutant presenilins specifically elevate the levels of the 42 residue beta-amyloid peptide in vivo: evidence for augmentation of a 42-specific gamma secretase. Hum. Mol. Genet. 13, 159–170. doi: 10.1093/hmg/ddh019, PMID: 14645205

[ref52] Jantzen L. Dumontoy S. Ramadan B. Houdayer C. Haffen E. Hichami A.. (2024). Dietary linoleic acid supplementation protects against obesity-induced microglial reactivity in mice. Sci. Rep. 14:6644. doi: 10.1038/s41598-024-56959-6, PMID: 38503857 PMC10951280

[ref53] Jensen T. L. Kiersgaard M. K. Sørensen D. B. Mikkelsen L. F. (2013). Fasting of mice: a review. Lab. Anim. 47, 225–240. doi: 10.1177/0023677213501659, PMID: 24025567

[ref54] Kantarci K. Weigand S. D. Petersen R. C. Boeve B. F. Knopman D. S. Gunter J.. (2007). Longitudinal 1H MRS changes in mild cognitive impairment and Alzheimer's disease. Neurobiol. Aging 28, 1330–1339. doi: 10.1016/j.neurobiolaging.2006.06.018, PMID: 16860440 PMC2766807

[ref55] Kao Y. C. Ho P. C. Tu Y. K. Jou I. M. Tsai K. J. (2020). Lipids and Alzheimer's disease. Int. J. Mol. Sci. 21:21. doi: 10.3390/ijms21041505, PMID: 32098382 PMC7073164

[ref56] Kim O. Y. Song J. (2024). Important roles of linoleic acid and α-linolenic acid in regulating cognitive impairment and neuropsychiatric issues in metabolic-related dementia. Life Sci. 337:122356. doi: 10.1016/j.lfs.2023.122356, PMID: 38123015

[ref57] Klunk W. E. Xu C. Panchalingam K. McClure R. J. Pettegrew J. W. (1996). Quantitative 1H and 31P MRS of PCA extracts of postmortem Alzheimer's disease brain. Neurobiol. Aging 17, 349–357. doi: 10.1016/0197-4580(96)00035-8, PMID: 8725895

[ref58] Koal T. Klavins K. Seppi D. Kemmler G. Humpel C. (2015). Sphingomyelin SM(d18:1/18:0) is significantly enhanced in cerebrospinal fluid samples dichotomized by pathological amyloid-β42, tau, and phospho-tau-181 levels. J. Alzheimers Dis. 44, 1193–1201. doi: 10.3233/jad-142319, PMID: 25408209 PMC4699259

[ref59] Kosicek M. Hecimovic S. (2013). Phospholipids and Alzheimer's disease: alterations, mechanisms and potential biomarkers. Int. J. Mol. Sci. 14, 1310–1322. doi: 10.3390/ijms14011310, PMID: 23306153 PMC3565322

[ref60] Kosicek M. Zetterberg H. Andreasen N. Peter-Katalinic J. Hecimovic S. (2012). Elevated cerebrospinal fluid sphingomyelin levels in prodromal Alzheimer's disease. Neurosci. Lett. 516, 302–305. doi: 10.1016/j.neulet.2012.04.019, PMID: 22521584

[ref61] Kothari V. Luo Y. Tornabene T. O'Neill A. M. Greene M. W. Geetha T.. (2017). High fat diet induces brain insulin resistance and cognitive impairment in mice. Biochim. Biophys. Acta Mol. basis Dis. 1863, 499–508. doi: 10.1016/j.bbadis.2016.10.006, PMID: 27771511

[ref62] Laske C. Leyhe T. Stransky E. Hoffmann N. Fallgatter A. J. Dietzsch J. (2011). Identification of a blood-based biomarker panel for classification of Alzheimer's disease. Int. J. Neuropsychopharmacol. 14, 1147–1155. doi: 10.1017/s1461145711000459, PMID: 21466745

[ref63] Li T. Su Q. Zhang Z. Zhang Y. Yang M. Wang Z.. (2022). Ube2c-inhibition alleviated amyloid pathology and memory deficits in APP/PS1 mice model of AD. Prog. Neurobiol. 215:102298. doi: 10.1016/j.pneurobio.2022.102298, PMID: 35671859

[ref64] Li X. Zhang Y. Ding X. Jin Y. Wei C. Xu J. (2023). Mass spectrometry chromatography-based metabolomics: the effect of long-term aerobic exercise on learning ability and the metabolism of intestinal contents in mice with Alzheimer's disease. Meta 13:1150. doi: 10.3390/metabo13111150, PMID: 37999246 PMC10673277

[ref65] Lindon J. C. Holmes E. Nicholson J. K. (2004). Metabonomics and its role in drug development and disease diagnosis. Expert. Rev. Mol. Diagn. 4, 189–199. doi: 10.1586/14737159.4.2.189 14995905

[ref66] Liu X. Dong T. Zhou Y. Huang N. Lei X. (2016). Exploring the binding proteins of glycolipids with bifunctional chemical probes. Angew. Chem. Int. Ed. Engl. 55, 14330–14334. doi: 10.1002/anie.201608827, PMID: 27762087

[ref67] Liu L. W. Yue H. Y. Zou J. Tang M. Zou F. M. Li Z. L.. (2022). Comprehensive metabolomics and lipidomics profiling uncovering neuroprotective effects of *Ginkgo biloba* L. leaf extract on Alzheimer's disease. Front. Pharmacol. 13:1076960. doi: 10.3389/fphar.2022.1076960, PMID: 36618950 PMC9810818

[ref68] Lowry J. R. Marshall N. Wenzel T. J. Murray T. E. Klegeris A. (2020). The dietary fatty acids α-linolenic acid (ALA) and linoleic acid (LA) selectively inhibit microglial nitric oxide production. Mol. Cell. Neurosci. 109:103569. doi: 10.1016/j.mcn.2020.103569, PMID: 33161065

[ref69] Maccioni R. B. Muñoz J. P. Barbeito L. (2001). The molecular bases of Alzheimer's disease and other neurodegenerative disorders. Arch. Med. Res. 32, 367–381. doi: 10.1016/s0188-4409(01)00316-2, PMID: 11578751

[ref70] Malm T. Koistinaho J. Kanninen K. (2011). Utilization of APPswe/PS1dE9 transgenic mice in research of Alzheimer's disease: focus on gene therapy and cell-based therapy applications. Int. J. Alzheimers Dis. 2011:517160. doi: 10.4061/2011/517160, PMID: 22114743 PMC3205616

[ref71] Marin R. Diaz M. (2018). Estrogen interactions with lipid rafts related to neuroprotection. Impact of brain ageing and menopause. Front. Neurosci. 12:128. doi: 10.3389/fnins.2018.00128, PMID: 29559883 PMC5845729

[ref72] Merrill A. H. Jr. (2011). Sphingolipid and glycosphingolipid metabolic pathways in the era of sphingolipidomics. Chem. Rev. 111, 6387–6422. doi: 10.1021/cr2002917, PMID: 21942574 PMC3191729

[ref73] Mesa-Herrera F. Taoro-González L. Valdés-Baizabal C. Diaz M. Marín R. (2019). Lipid and lipid raft alteration in aging and neurodegenerative diseases: a window for the development of new biomarkers. Int. J. Mol. Sci. 20:20. doi: 10.3390/ijms20153810, PMID: 31382686 PMC6696273

[ref74] Mielke M. M. Lyketsos C. G. (2006). Lipids and the pathogenesis of Alzheimer's disease: is there a link? Int. Rev. Psychiatry 18, 173–186. doi: 10.1080/09540260600583007, PMID: 16777671

[ref75] Morris M. C. Evans D. A. Bienias J. L. Tangney C. C. Bennett D. A. Aggarwal N.. (2003). Dietary fats and the risk of incident Alzheimer disease. Arch. Neurol. 60, 194–200. doi: 10.1001/archneur.60.2.194, PMID: 12580703

[ref76] Morselli E. Santos R. S. Gao S. Ávalos Y. Criollo A. Palmer B. F.. (2018). Impact of estrogens and estrogen receptor-α in brain lipid metabolism. Am. J. Physiol. Endocrinol. Metab. 315, E7–e14. doi: 10.1152/ajpendo.00473.2017, PMID: 29509437 PMC7717113

[ref77] Peña-Bautista C. Álvarez-Sánchez L. Roca M. García-Vallés L. Baquero M. Cháfer-Pericás C. (2022). Plasma Lipidomics approach in early and specific Alzheimer's disease diagnosis. J. Clin. Med. 11:11. doi: 10.3390/jcm11175030, PMID: 36078960 PMC9457360

[ref78] Perez S. E. Berg B. M. Moore K. A. He B. Counts S. E. Fritz J. J.. (2010). DHA diet reduces AD pathology in young APPswe/PS1 Delta E9 transgenic mice: possible gender effects. J. Neurosci. Res. 88, 1026–1040. doi: 10.1002/jnr.22266, PMID: 19859965 PMC3118087

[ref79] Pifferi F. Dorieux O. Castellano C. A. Croteau E. Masson M. Guillermier M.. (2015). Long-chain n-3 PUFAs from fish oil enhance resting state brain glucose utilization and reduce anxiety in an adult nonhuman primate, the grey mouse lemur. J. Lipid Res. 56, 1511–1518. doi: 10.1194/jlr.M058933, PMID: 26063461 PMC4513992

[ref80] Pripp A. H. (2018). Pearson's or spearman's correlation coefficients. Tidsskr. Nor. Laegeforen. 138. doi: 10.4045/tidsskr.18.0042, PMID: 29737766

[ref81] Proitsi P. Kim M. Whiley L. Simmons A. Sattlecker M. Velayudhan L.. (2017). Association of blood lipids with Alzheimer's disease: a comprehensive lipidomics analysis. Alzheimers Dement. 13, 140–151. doi: 10.1016/j.jalz.2016.08.003, PMID: 27693183

[ref82] Qiang Y. X. You J. He X. Y. Guo Y. Deng Y. T. Gao P. Y.. (2024). Plasma metabolic profiles predict future dementia and dementia subtypes: a prospective analysis of 274,160 participants. Alzheimers Res. Ther. 16:16. doi: 10.1186/s13195-023-01379-3, PMID: 38254212 PMC10802055

[ref83] Radde R. Bolmont T. Kaeser S. A. Coomaraswamy J. Lindau D. Stoltze L.. (2006). Abeta42-driven cerebral amyloidosis in transgenic mice reveals early and robust pathology. EMBO Rep. 7, 940–946. doi: 10.1038/sj.embor.7400784, PMID: 16906128 PMC1559665

[ref84] Sastry P. S. (1985). Lipids of nervous tissue: composition and metabolism. Prog. Lipid Res. 24, 69–176. doi: 10.1016/0163-7827(85)90011-6, PMID: 3916238

[ref85] Selkoe D. J. (2003). Folding proteins in fatal ways. Nature 426, 900–904. doi: 10.1038/nature02264, PMID: 14685251

[ref86] Selkoe D. J. Schenk D. (2003). Alzheimer's disease: molecular understanding predicts amyloid-based therapeutics. Annu. Rev. Pharmacol. Toxicol. 43, 545–584. doi: 10.1146/annurev.pharmtox.43.100901.140248, PMID: 12415125

[ref87] Shen L. Han B. Geng Y. Wang J. Wang Z. Wang M. (2017). Amelioration of cognitive impairments in APPswe/PS1dE9 mice is associated with metabolites alteration induced by total salvianolic acid. PLoS One 12:e0174763. doi: 10.1371/journal.pone.0174763, PMID: 28358909 PMC5373599

[ref88] Shen S. Yang L. Li L. Bai Y. Cai C. Liu H. (2017). A plasma lipidomics strategy reveals perturbed lipid metabolic pathways and potential lipid biomarkers of human colorectal cancer. J. Chromatogr. B Analyt. Technol. Biomed. Life Sci. 1068-1069, 41–48. doi: 10.1016/j.jchromb.2017.10.004, PMID: 29028617

[ref89] Sinclair A. J. (2019). Docosahexaenoic acid and the brain- what is its role? Asia Pac. J. Clin. Nutr. 28, 675–688. doi: 10.6133/apjcn.201912_28(4).0002 31826363

[ref90] Smith C. A. Want E. J. O'Maille G. Abagyan R. Siuzdak G. (2006). XCMS: processing mass spectrometry data for metabolite profiling using nonlinear peak alignment, matching, and identification. Anal. Chem. 78, 779–787. doi: 10.1021/ac051437y, PMID: 16448051

[ref91] Söderberg M. Edlund C. Kristensson K. Dallner G. (1990). Lipid compositions of different regions of the human brain during aging. J. Neurochem. 54, 415–423. doi: 10.1111/j.1471-4159.1990.tb01889.x, PMID: 2299344

[ref92] Stapleton M. Kubaski F. Mason R. W. Shintaku H. Kobayashi H. Yamaguchi S.. (2020). Newborn screening for mucopolysaccharidoses: measurement of glycosaminoglycans by LC-MS/MS. Mol Genet Metab Rep 22:100563. doi: 10.1016/j.ymgmr.2019.100563, PMID: 31956510 PMC6957835

[ref93] Strnad Š. Pražienková V. Sýkora D. Cvačka J. Maletínská L. Popelová A.. (2019). The use of 1,5-diaminonaphthalene for matrix-assisted laser desorption/ionization mass spectrometry imaging of brain in neurodegenerative disorders. Talanta 201, 364–372. doi: 10.1016/j.talanta.2019.03.117, PMID: 31122436

[ref94] Sun R. Lin Z. Wang X. Liu L. Huo M. Zhang R.. (2022). Correction: AADAC protects colorectal cancer liver colonization from ferroptosis through SLC7A11-dependent inhibition of lipid peroxidation. J. Exp. Clin. Cancer Res. 41:313. doi: 10.1186/s13046-022-02508-w, PMID: 36163032 PMC9511737

[ref95] Svennerholm L. Boström K. Jungbjer B. Olsson L. (1994). Membrane lipids of adult human brain: lipid composition of frontal and temporal lobe in subjects of age 20 to 100 years. J. Neurochem. 63, 1802–1811. doi: 10.1046/j.1471-4159.1994.63051802.x, PMID: 7931336

[ref96] Tajima Y. Ishikawa M. Maekawa K. Murayama M. Senoo Y. Nishimaki-Mogami T.. (2013). Lipidomic analysis of brain tissues and plasma in a mouse model expressing mutated human amyloid precursor protein/tau for Alzheimer's disease. Lipids Health Dis. 12:68. doi: 10.1186/1476-511x-12-68, PMID: 23659495 PMC3668217

[ref97] Toledo J. B. Arnold M. Kastenmüller G. Chang R. Baillie R. A. Han X.. (2017). Metabolic network failures in Alzheimer's disease: a biochemical road map. Alzheimers Dement. 13, 965–984. doi: 10.1016/j.jalz.2017.01.020, PMID: 28341160 PMC5866045

[ref98] Touboul D. Gaudin M. (2014). Lipidomics of Alzheimer's disease. Bioanalysis 6, 541–561. doi: 10.4155/bio.13.346, PMID: 24568356

[ref99] Turri M. Conti E. Pavanello C. Gastoldi F. Palumbo M. Bernini F.. (2023). Plasma and cerebrospinal fluid cholesterol esterification is hampered in Alzheimer's disease. Alzheimers Res. Ther. 15:95. doi: 10.1186/s13195-023-01241-6, PMID: 37210544 PMC10199596

[ref100] van Ginneken V. Verhey E. Poelmann R. Ramakers R. van Dijk K. W. Ham L.. (2007). Metabolomics (liver and blood profiling) in a mouse model in response to fasting: a study of hepatic steatosis. Biochim. Biophys. Acta 1771, 1263–1270. doi: 10.1016/j.bbalip.2007.07.007, PMID: 17904417

[ref101] Varma V. R. Oommen A. M. Varma S. Casanova R. An Y. Andrews R. M.. (2018). Brain and blood metabolite signatures of pathology and progression in Alzheimer disease: a targeted metabolomics study. PLoS Med. 15:e1002482. doi: 10.1371/journal.pmed.1002482, PMID: 29370177 PMC5784884

[ref102] Wang Y. Y. Sun Y. P. Luo Y. M. Peng D. H. Li X. Yang B. Y.. (2021). Biomarkers for the clinical diagnosis of Alzheimer's disease: metabolomics analysis of brain tissue and blood. Front. Pharmacol. 12:700587. doi: 10.3389/fphar.2021.700587, PMID: 34366852 PMC8333692

[ref103] Wang Z. Zhang D. Cheng C. Lin Z. Zhou D. Sun Y.. (2023). Supplementation of medium-chain triglycerides combined with docosahexaenoic acid inhibits amyloid Beta protein deposition by improving brain glucose metabolism in APP/PS1 mice. Nutrients 15:15. doi: 10.3390/nu15194244, PMID: 37836528 PMC10574179

[ref104] Wei R. Wang J. Su M. Jia E. Chen S. Chen T.. (2018). Missing value imputation approach for mass spectrometry-based metabolomics data. Sci. Rep. 8:663. doi: 10.1038/s41598-017-19120-0, PMID: 29330539 PMC5766532

[ref105] Whelan J. Fritsche K. (2013). Linoleic acid. Adv. Nutr. 4, 311–312. doi: 10.3945/an.113.003772, PMID: 23674797 PMC3650500

[ref106] Whiley L. Sen A. Heaton J. Proitsi P. García-Gómez D. Leung R.. (2014). Evidence of altered phosphatidylcholine metabolism in Alzheimer's disease. Neurobiol. Aging 35, 271–278. doi: 10.1016/j.neurobiolaging.2013.08.001, PMID: 24041970 PMC5866043

[ref107] Wiklund S. Johansson E. Sjöström L. Mellerowicz E. J. Edlund U. Shockcor J. P.. (2008). Visualization of GC/TOF-MS-based metabolomics data for identification of biochemically interesting compounds using OPLS class models. Anal. Chem. 80, 115–122. doi: 10.1021/ac0713510, PMID: 18027910

[ref108] Xiong H. Callaghan D. Wodzinska J. Xu J. Premyslova M. Liu Q. Y.. (2011). Biochemical and behavioral characterization of the double transgenic mouse model (APPswe/PS1dE9) of Alzheimer's disease. Neurosci. Bull. 27, 221–232. doi: 10.1007/s12264-011-1015-7, PMID: 21788993 PMC5560305

[ref109] Yao J. K. Wengenack T. M. Curran G. L. Poduslo J. F. (2009). Reduced membrane lipids in the cortex of Alzheimer's disease transgenic mice. Neurochem. Res. 34, 102–108. doi: 10.1007/s11064-008-9673-1, PMID: 18373196

[ref110] Yokoyama M. Kobayashi H. Tatsumi L. Tomita T. (2022). Mouse models of Alzheimer's disease. Front. Mol. Neurosci. 15:912995. doi: 10.3389/fnmol.2022.912995, PMID: 35799899 PMC9254908

[ref111] Zhang Z. H. Cao X. C. Peng J. Y. Huang S. L. Chen C. Jia S. Z.. (2022). Reversal of lipid metabolism dysregulation by selenium and folic acid co-supplementation to mitigate pathology in Alzheimer's disease. Antioxidants (Basel) 11:829. doi: 10.3390/antiox11050829, PMID: 35624693 PMC9138008

[ref112] Zhang Q. Li Y. Sui P. Sun X. H. Gao Y. Wang C. Y. (2024). MALDI mass spectrometry imaging discloses the decline of sulfoglycosphingolipid and glycerophosphoinositol species in the brain regions related to cognition in a mouse model of Alzheimer's disease. Talanta 266:125022. doi: 10.1016/j.talanta.2023.125022, PMID: 37619472

[ref113] Zhang X. Liu W. Zan J. Wu C. Tan W. (2020). Untargeted lipidomics reveals progression of early Alzheimer's disease in APP/PS1 transgenic mice. Sci. Rep. 10:14509. doi: 10.1038/s41598-020-71510-z, PMID: 32884056 PMC7471266

[ref114] Zhang A. H. Ma Z. M. Kong L. Gao H. L. Sun H. Wang X. Q.. (2020). High-throughput lipidomics analysis to discover lipid biomarkers and profiles as potential targets for evaluating efficacy of Kai-Xin-san against APP/PS1 transgenic mice based on UPLC-Q/TOF-MS. Biomed. Chromatogr. 34:e4724. doi: 10.1002/bmc.4724, PMID: 31755117

[ref115] Zhou C. N. Chao F. L. Zhang Y. Jiang L. Zhang L. Fan J. H.. (2019). Fluoxetine delays the cognitive function decline and synaptic changes in a transgenic mouse model of early Alzheimer's disease. J. Comp. Neurol. 527, 1378–1387. doi: 10.1002/cne.24616, PMID: 30592045

