# Supplementary figures and images for: Quantitative analysis of targeted lipidomics in the hippocampus of APP/PS1 mice employing the UHPLC-MS/MS method
Source: Front Aging Neurosci. 2025 Jul 7;17:1561831. doi: 10.3389/fnagi.2025.1561831 (PMC12277340; doi:10.3389/fnagi.2025.1561831)

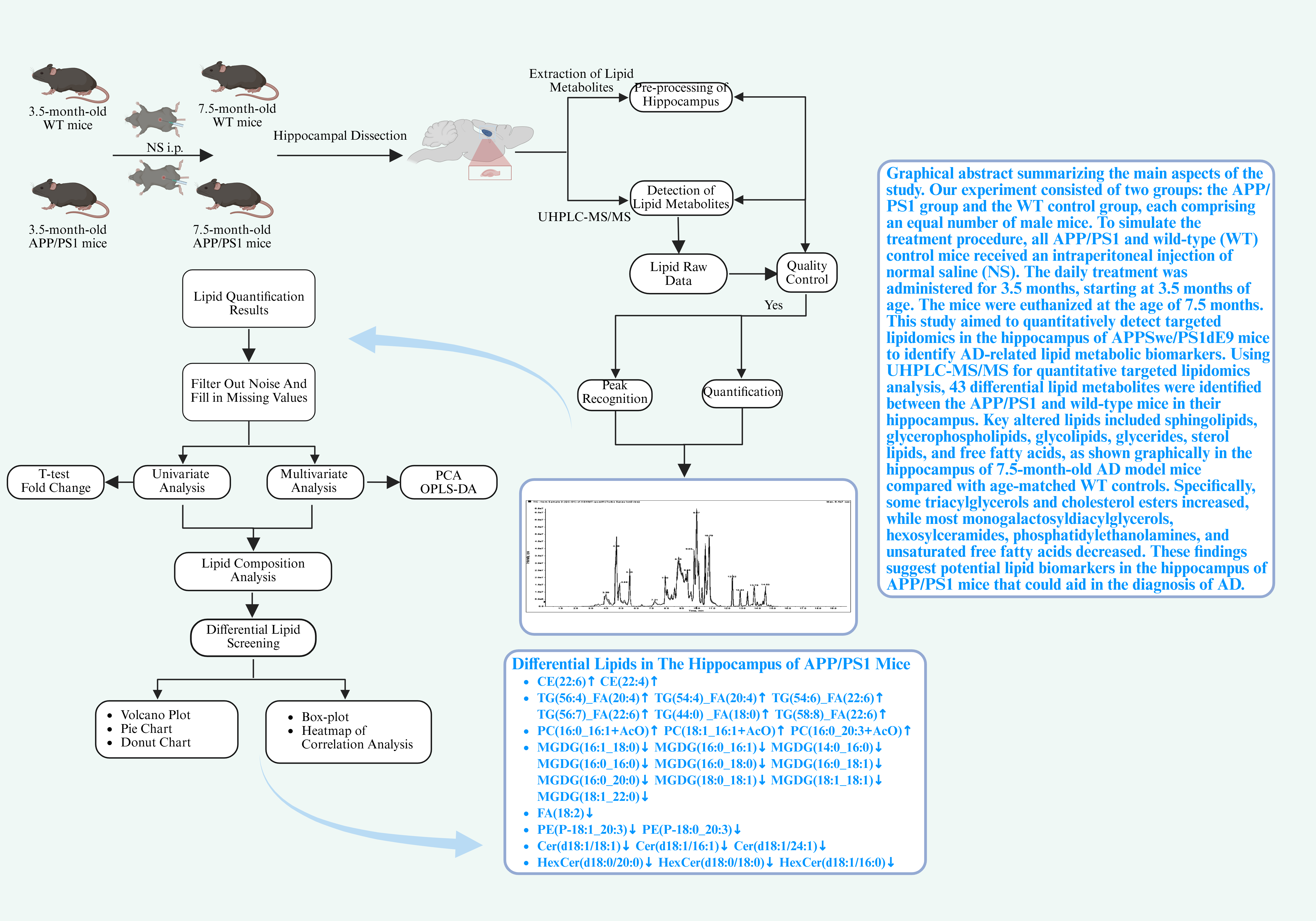

Supplement: Supplementary file 1 [file Image_1.jpeg]

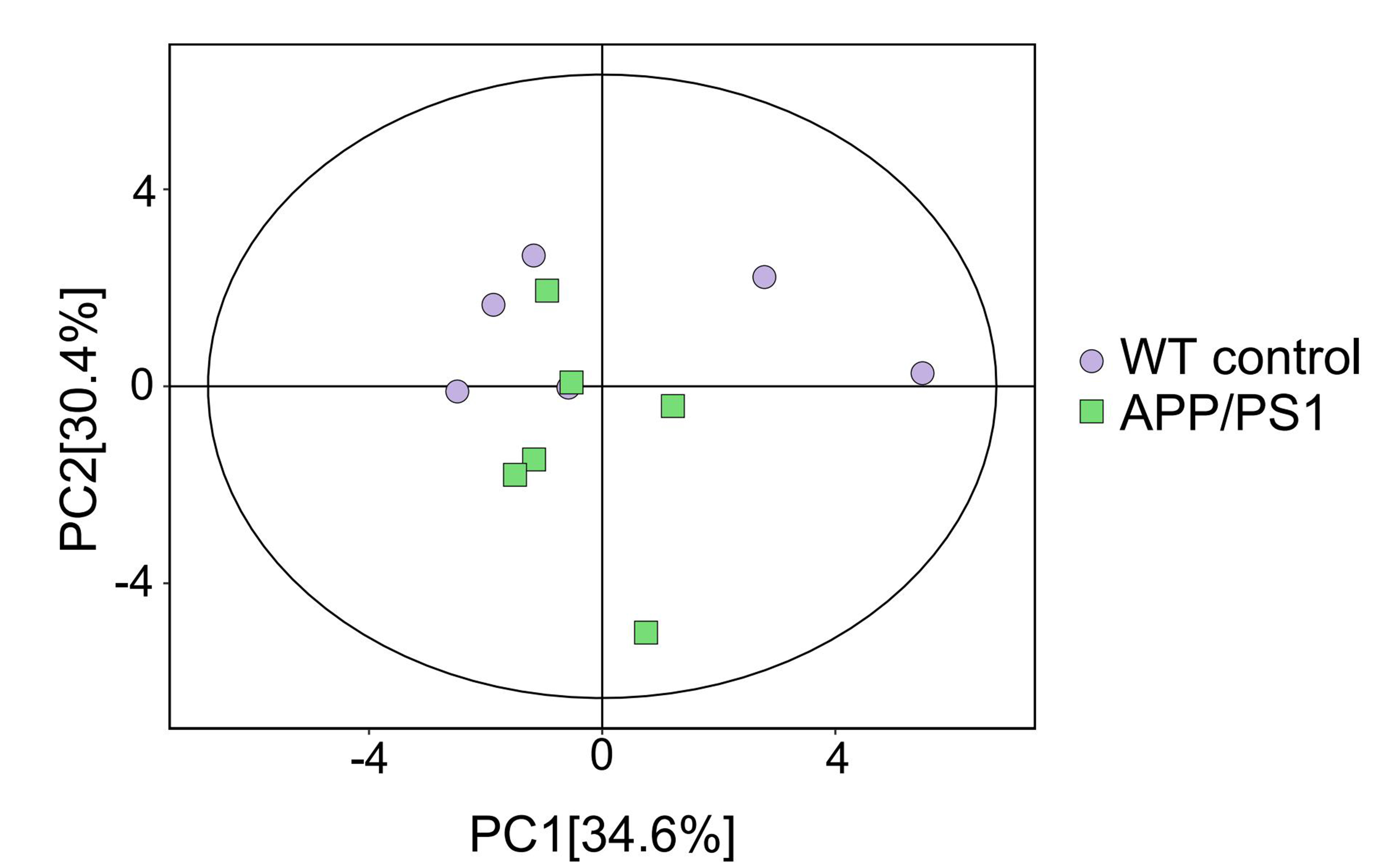

Supplement: Supplementary file 2 [file Image_2.jpeg]
